# Convergent Alterations of a Protein Hub Produce Divergent Effects within a Binding Site

**DOI:** 10.1021/acschembio.2c00273

**Published:** 2022-05-25

**Authors:** Ali Imran, Brandon S. Moyer, Dan Kalina, Thomas M. Duncan, Kelsey J. Moody, Aaron J. Wolfe, Michael S. Cosgrove, Liviu Movileanu

**Affiliations:** † Department of Physics, Syracuse University, 201 Physics Building, Syracuse, New York 13244-1130, United States; ‡ Ichor Life Sciences, Inc., 2651 US Route 11, LaFayette, New York 13084, United States; § Department of Chemistry, State University of New York College of Environmental Science and Forestry, 1 Forestry Dr., Syracuse, New York 13210, United States; ∥ Department of Biochemistry and Molecular Biology, State University of New York Upstate Medical University, 4249 Weiskotten Hall, 766 Irving Avenue, Syracuse, New York 13210, United States; ⊥ Lewis School of Health Sciences, Clarkson University, 8 Clarkson Avenue, Potsdam, New York 13699, United States; # Department of Biomedical and Chemical Engineering, Syracuse University, 329 Link Hall, Syracuse, New York 13244, United States; ¶ The BioInspired Institute, Syracuse University, Syracuse, New York 13244, United States

## Abstract

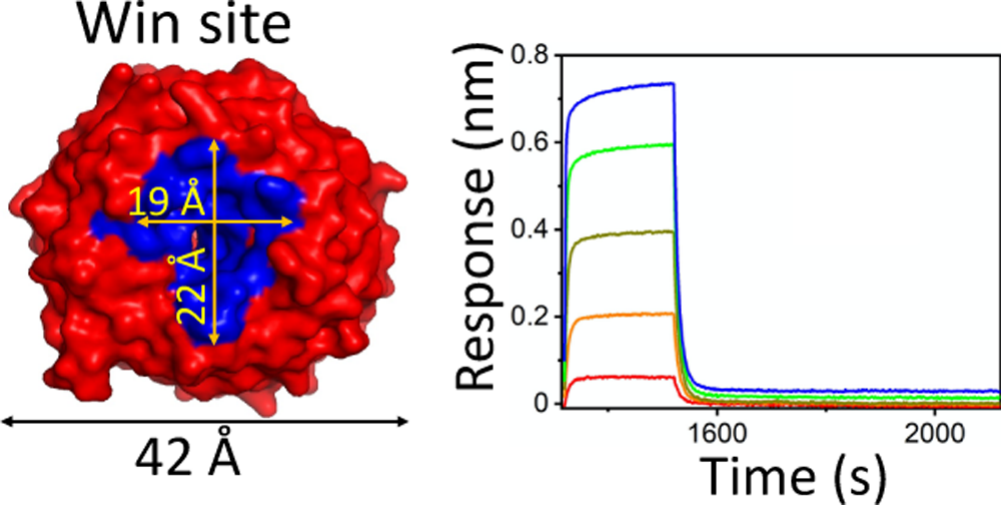

Progress in tumor sequencing and cancer databases has created an enormous amount of information that scientists struggle to sift through. While several research groups have created computational methods to analyze these databases, much work still remains in distinguishing key implications of pathogenic mutations. Here, we describe an approach to identify and evaluate somatic cancer mutations of WD40 repeat protein 5 (WDR5), a chromatin-associated protein hub. This multitasking protein maintains the functional integrity of large multi-subunit enzymatic complexes of the six human SET1 methyltransferases. Remarkably, the somatic cancer mutations of WDR5 preferentially distribute within and around an essential cavity, which hosts the WDR5 interaction (Win) binding site. Hence, we assessed the real-time binding kinetics of the interactions of key clustered WDR5 mutants with the Win motif peptide ligands of the SET1 family members (SET1_Win_). Our measurements highlight that this subset of mutants exhibits divergent perturbations in the kinetics and strength of interactions not only relative to those of the native WDR5 but also among various SET1_Win_ ligands. These outcomes could form a fundamental basis for future drug discovery and other developments in medical biotechnology.

## Introduction

**Figure 1:**
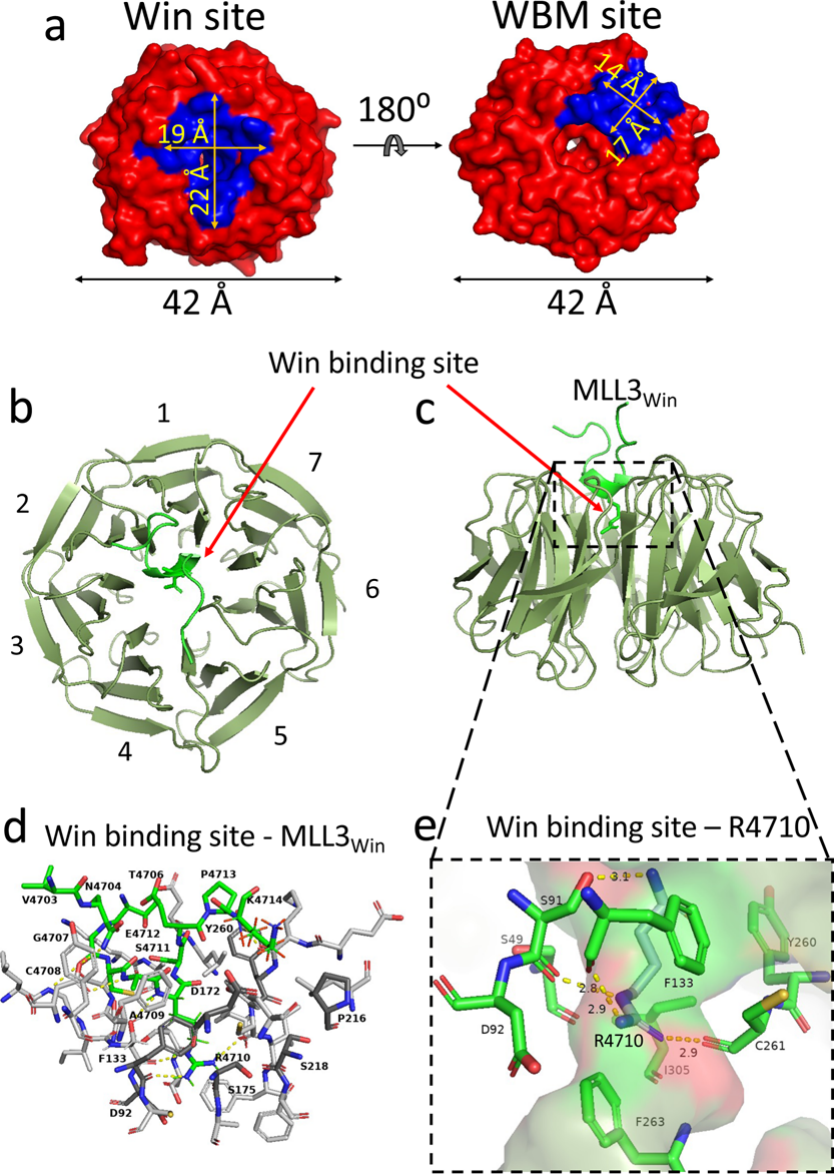
Two binding sites of WDR5 and the structure of the WDR5-MLL3_Win_ complex. (a) Representations of the Win and WBM binding sites of WDR5. Orientations of WDR5 in the two cartoons are 180° with respect to each other. (b) Top view of the WDR5-MLL3_Win_ complex. (c) Side view of the WDR5-MLL3_Win_ complex. (d) Side view of the interaction sites between MLL3_Win_ (green) and WDR5 (gray). All presented residues are within 5 Å of the other binding partner. The residues corresponding to the WDR5 mutations explored in this study are marked in dark gray. MLL3_Win_ residues are labeled as well. Potential hydrogen bonds between the two binding partners are shown as yellow dotted lines. (e) Key residues of the WDR5 binding cavity involved in hydrogen bonding with the evolutionarily conserved Arg residue (R4710) of MLL3_Win_ at position P_0_ (Table S1). The hydrogen bonds are indicated by thick dashed lines marked in yellow. The cutoff distance for identifying these hydrogen bonds was 4.0 Å. WDR5 was represented using pdb entry 4ERY.^35^

WD40 repeat proteins (WDRs) are among the most abundant protein–protein interaction domains in the human proteome.^1−3^ WDRs are either implicated in numerous cell signaling pathways^4,5^ or in scaffolding large multi-subunit enzymatic complexes.^6,7^ Notably, WD40 repeat protein 5 (WDR5) is a highly conserved nuclear hub, which is primarily known for its regulatory role in histone 3 lysine 4 (H3K4) mono- and di-methylation.^8−13^ In this process, WDR5 bridges the interaction between the catalytic domain of mixed lineage leukemia MLL/SET1 family proteins and other subunits of the large methyltransferase complex. The assembly and stability of this enzymatic complex is necessary for optimal methyltransferase activity.^14−16^ In addition, WDR5 interacts with other protein partners, such as transcription factor MYC^17−20^ and 3-phosphoinositide-dependent protein kinase 1 (PDPK1).^21^ Two highly conserved motifs of these protein binders, the WDR5 interaction (Win) motif^22−24^ and WDR5-binding motif (WBM),^18,25,26^ are deemed responsible for the vast majority of their interactions with WDR5. Interactions corresponding to these motifs are mediated by the Win and WBM sites, respectively (Figure 1a).

For oncoproteins, the driver cancer mutations preferentially populate either within an active site or on their binding surface.^27,28^ Based upon this argument, we postulated that missense somatic cancer mutations of WDR5 form a dense cluster either within one or both binding sites. Databases, such as Catalogue of Somatic Mutations in Cancer (COSMIC),^29,30^ have become instrumental resources for unraveling the influential roles of specific proteins in different cancers.^31−33^ However, by determining the density and location of known mutations, their important subsets under disease-like conditions can potentially be identified. Using the clustering of mutations in protein structures (CLUMPS) method,^34^ we were able to determine, in accord with our hypothesis, that the high-density distribution of WDR5 missense alterations occurs within and around the Win binding site.

The Win binding site is located within a central cavity and facilitates high-affinity interactions of WDR5 with each of the six human histone methyltransferases (HMTs; MLL1-4 and SETd1A-B), participating in the formation of corresponding six SET1 enzymatic complexes.^35,36^ Rearrangements in the MLL1 gene lead to solid tumors and aggressive lymphocytic leukemias in humans.^37^ Moreover, WDR5 is overexpressed under various oncogenic conditions and its upregulation catalyzes cancer development.^38−42^ In recent years, the multitasking Win binding site has received a lot of interest^21,43−45^ because it is a promising target for anti-cancer drug discovery.^46−53^ Therefore, a quantitative understanding of WDR5’s interactions with other Win motif partners has wide-ranging fundamental significance.^50,54−57^ For example, the kinetic fingerprints and affinities of the interactions of WDR5 with Win motif peptides of SET1 family members (SET1_Win_) have been previously reported.^35,36^

Stimulated by our finding using the CLUMPS method,^34^ we explored the impact of somatic cancer mutations of WDR5 on its interactions with 14-residue SET1_Win_ peptide ligands of the six SET1 proteins (Figure 1b,c; Table S1). The WDR5-SET1 interaction requires the precise insertion of a highly conserved Arg residue of SET1 proteins into the Win binding site (Figure 1d,e).^23^ This key interaction is a prerequisite for the structural and functional integrity of the C-terminal catalytic domain of SET1 proteins.^15,22,23^ SET1_Win_ ligands recapitulate the native interactions of the six SET1 proteins with WDR5 through the Win binding site.^35,36^ Therefore, we utilized the benefit of biolayer interferometry (BLI)^44,58−60^ for high-throughput settings and immobilized these SET1_Win_ ligands onto the sensor surface. In this way, we probed the real-time kinetics and dynamics of their interactions with a subset of these WDR5 mutants, whose missense alterations are located within and around the Win binding site. Remarkably, while these clustered mutations feature spatial proximity, they exhibit divergent effects on interactions with each of the six SET1_Win_ ligands. Finally, the results of this scalable kinetic platform were confirmed by orthogonal determinations of affinity constants of these interactions using steady-state fluorescence polarization (FP) spectroscopy.^61,62^

## Results and Discussion

### Use of CLUMPS for the Identification of Mutation Clustering in WDR5

We employed the CLUMPS method^34^ to investigate the three-dimensional (3D) clustering of 68 WDR5 mutations identified in 68 tumors. The missense mutations were comprehensively compiled using the COSMIC database.^29,30,63^ Information collected for each mutation included the residue number, the number of tumor samples, in which a certain mutation was noted, and the total number of mutations, *N*, in a tumor sample. *N* was used as a measure of the accumulation of genetic damage in a tumor sample, in which a certain mutation was sequenced. We hypothesize the lower the accumulated genetic damage in a tumor sample, the less noisy signature (e.g., populated by numerous insignificant mutations) would be in that sample. Hence, a mutation selected from a subset of mutations of that tumor sample exhibits an increased likelihood of being an important mutation. Four overlapping subsets of mutations were created from the total set of known mutations with the following conditions: *N* < 10 000, *N* < 5000, *N* < 1000, and *N* < 500 (Table 1). For each subset, we calculated a weighted average proximity (WAP) score and the corresponding *P*-value (Materials and Methods; Figure S1). We found that a subset of WDR5 mutations with a relatively low *N* (*N* < 500) is more likely to show mutation clustering because *P*-value was smaller than 0.03. Notably, the low-*N* subset also showed a substantial presence of mutations within and around the Win binding site (Tables S2–S3). Therefore, a subset of seven mutations was selected from all known WDR5 somatic cancer mutations within and around the Win binding site (Table S4). This approach allowed us to study the effects of these mutations on the kinetics and dynamics of WDR5-SET1_Win_ interactions.

**Table 1:** Results of Mutation Clustering of WDR5 for Different Subsets of *N*^a^

*N*	*m*	WAP score	*P*-value
<10 000	51	2.258	0.403
<5000	33	0.931	0.206
<1000	11	0.095	0.111
<500	8	0.072	0.025

a WAP scores were calculated using four different subsets of mutations divided on the basis of the genetic damage, *N*, in their corresponding tumors. The *P*-values were calculated by comparison to configurations with random permutations of the distribution of mutations. 10^6^ configurations were used for each subset. *N* is the total number of mutations in a given tumor sample. *m* is the total number of mutations that met the condition *N* < *N*_max_, where *N*_max_ is the upper limit of the number of mutations in a given tumor sample. *N*_max_ values are listed below for four data subsets on the first column. *m* was kept constant for all configurations of a subset.

### Biolayer Interferometry Measurements

**Figure 2:**
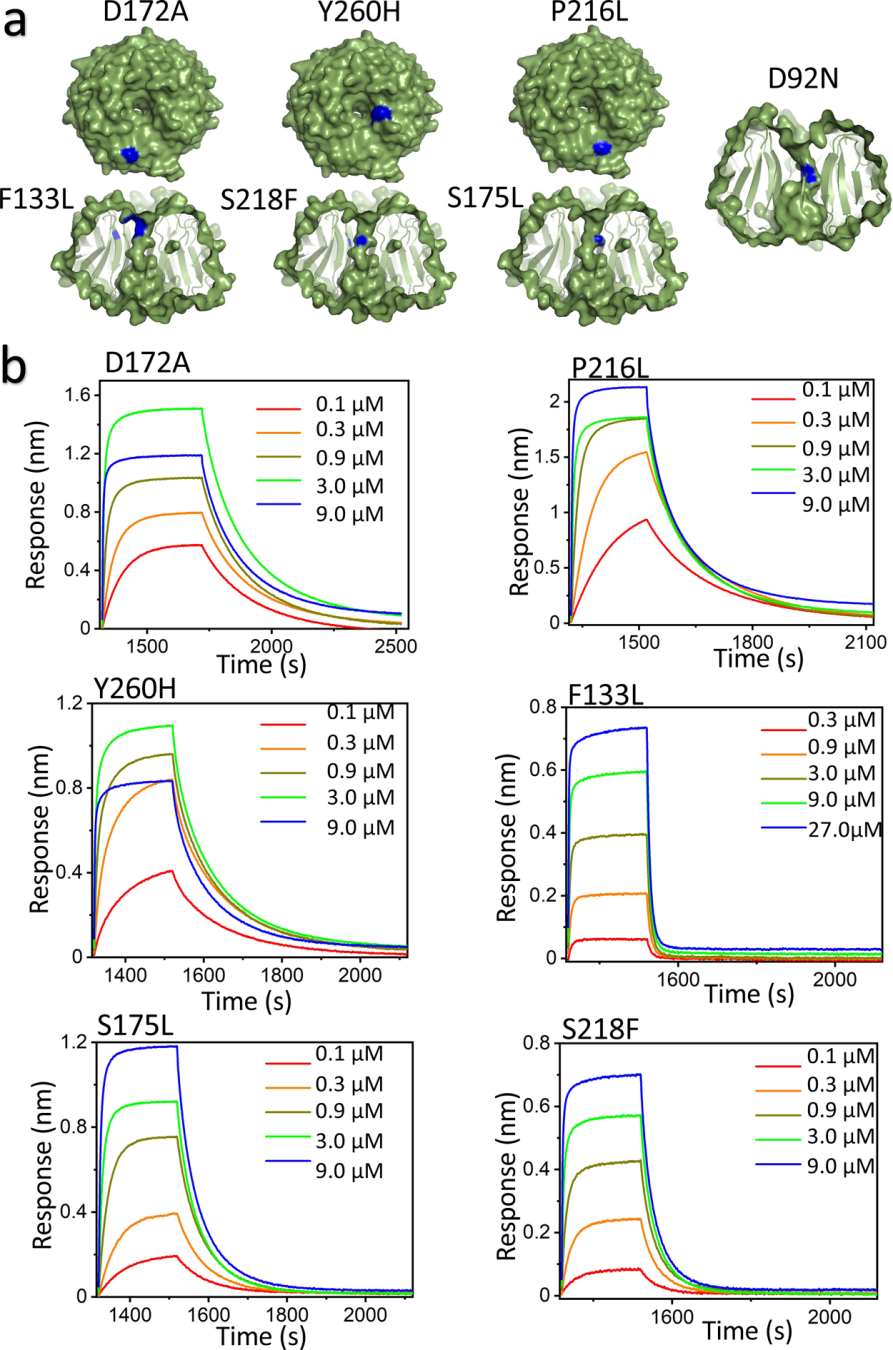
Label-free optical BLI sensorgrams of the WDR5 mutant-MLL3_Win_ interactions. (a) Locations of the surface and cavity WDR5 mutations are shown in blue using surface and cross-sectional views of WDR5, respectively. (b) BLI sensorgrams showing the association and dissociation phases. For each WDR5 mutation, sensors with immobilized MLL3_Win_ ligand were immersed in buffers containing different WDR5 concentrations (listed on sensorgrams) to monitor association kinetics. Sensors were then transferred to buffer alone to monitor dissociation kinetics.

In this study, targeted mutations have locations either within the WDR5 cavity (F133L, S175L, S218F, and D92N) or on the external surface and near the cavity (D172A, Y260H, and P216L) (Figure 2a; Table S4). These mutants were chosen based on their proximal locations to residues deemed to play key roles in SET1_Win_ interactions with the native WDR5 protein (Figure S2, Tables S5–S6).^22,23,35,36^ BLI measurements were used to determine the association (*k*_on_) and dissociation (*k*_off_) rate constants of WDR5-SET1_Win_ interactions.^58,59^ 14-residue SET1_Win_ peptide ligands, namely, MLL1_Win_, MLL2_Win_, MLL3_Win_, MLL4_Win_, SETd1A_Win_, and SETd1B_Win_, were biotinylated at the N terminus and amidated at the C-terminus (Table S1). A 9-residue Gly/Ser-rich peptide spacer was inserted between the biotinylated site and the SET1_Win_ sequence to avoid any steric hindrance of WDR5-SET1_Win_ interactions from the sensor surface. Biotinylated SET1_Win_ peptides were then tethered to the surface of streptavidin-coated sensors. Binding interactions of WDR5 with SET1_Win_ ligands attached to the sensor surface were monitored through changes in the optical interference pattern generated by reflected light waves at the sensor surface (Figure 2b). The association binding curves were acquired by placing the BLI sensors in distinct wells of varying WDR5 concentration. The dissociation binding curves were collected by placing the BLI sensors in wells containing WDR5-free buffer. It should be mentioned that all association and dissociation phases obeyed single-exponential fits, suggesting bimolecular association processes and unimolecular dissociation mechanisms of these binding phases, respectively.

Interestingly, we noted very weak binding interactions of all SET1_Win_ peptides with D92N, a cavity WDR5 mutant (Figures 2a and S3). While these interactions are detectable, they cannot be accurately quantified using BLI likely due to either a very low *k*_on_ or a very high *k*_off_, or both. A couple of possibilities could explain this interesting outcome. First, Asn-92 might interfere with the two hydrogen bonds between the Arg residue at the P_0_ position of the SET1_Win_ ligand (Table S1) and S91, a neighboring residue of WDR5 (Table S5). Second, the positively charged guanidinium group of Arg at P_0_ of SET1_Win_ might make an N–O salt bridge with the negatively charged carboxyl group of Asp-92 (Table S6).^64^ In addition, Asp-92 forms a salt bridge with Lys-52 located between β strands. Therefore, the absence of Asp-92 might alter the local conformation of the binding pocket.

### Surface Mutants

**Figure 3:**
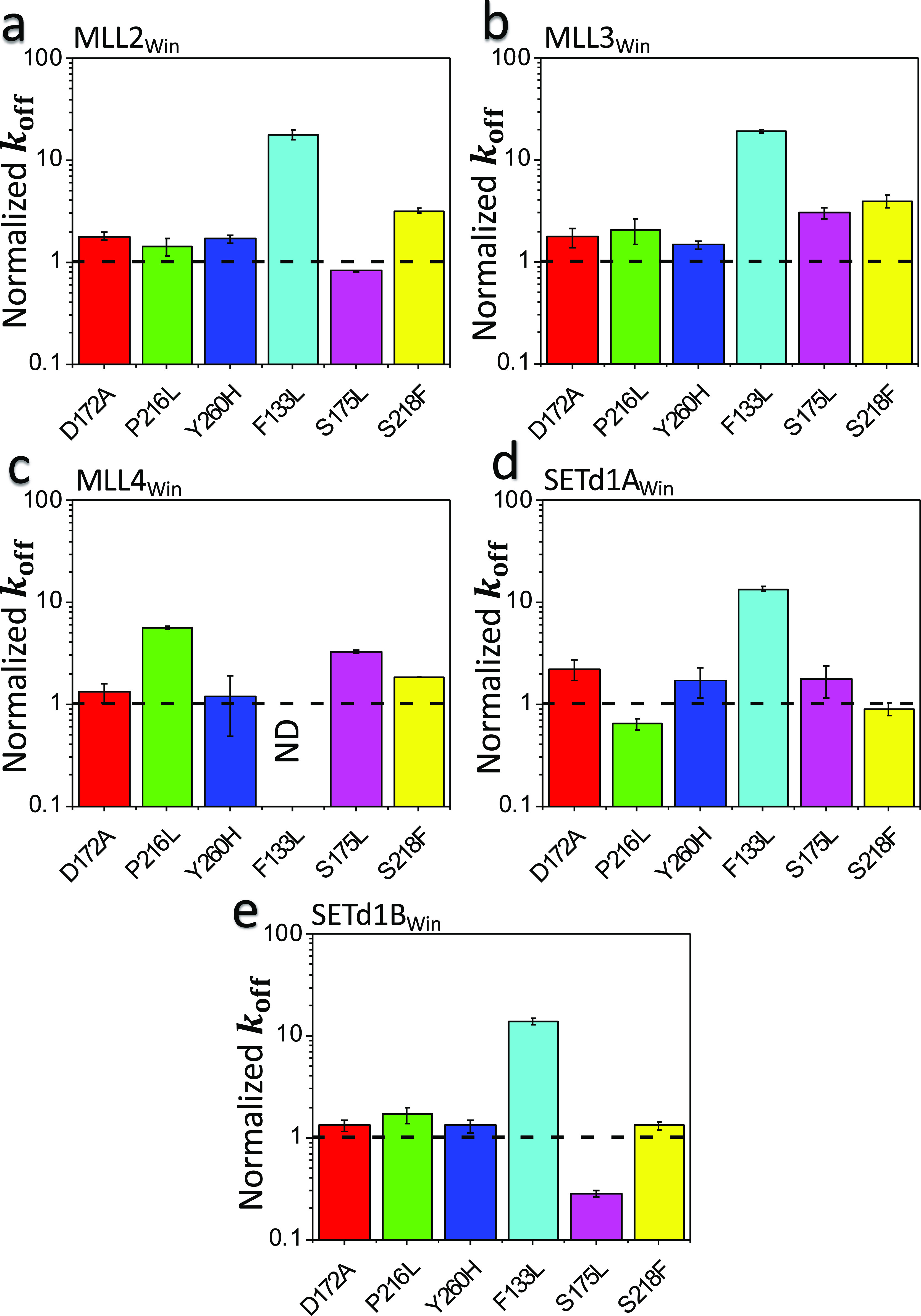
Normalized dissociation rate constants of the WDR5 mutant-SET1_Win_ interactions using BLI sensorgrams. The *k*_off_ values for each SET1_Win_ ligand’s interaction with mutants have been divided by the *k*_off_ of that SET1_Win_ ligand’s interaction with the native WDR5 protein. (a) MLL2_Win_, (b) MLL3_Win_, (c) MLL4_Win_, (d) SETd1A_Win_, and (e) SETd1B_Win_. ND stands for “Not Determined”. Using a BLI measurement, the interaction between F133L and MLL4_Win_ was detectable, but not quantifiable.

**Figure 4:**
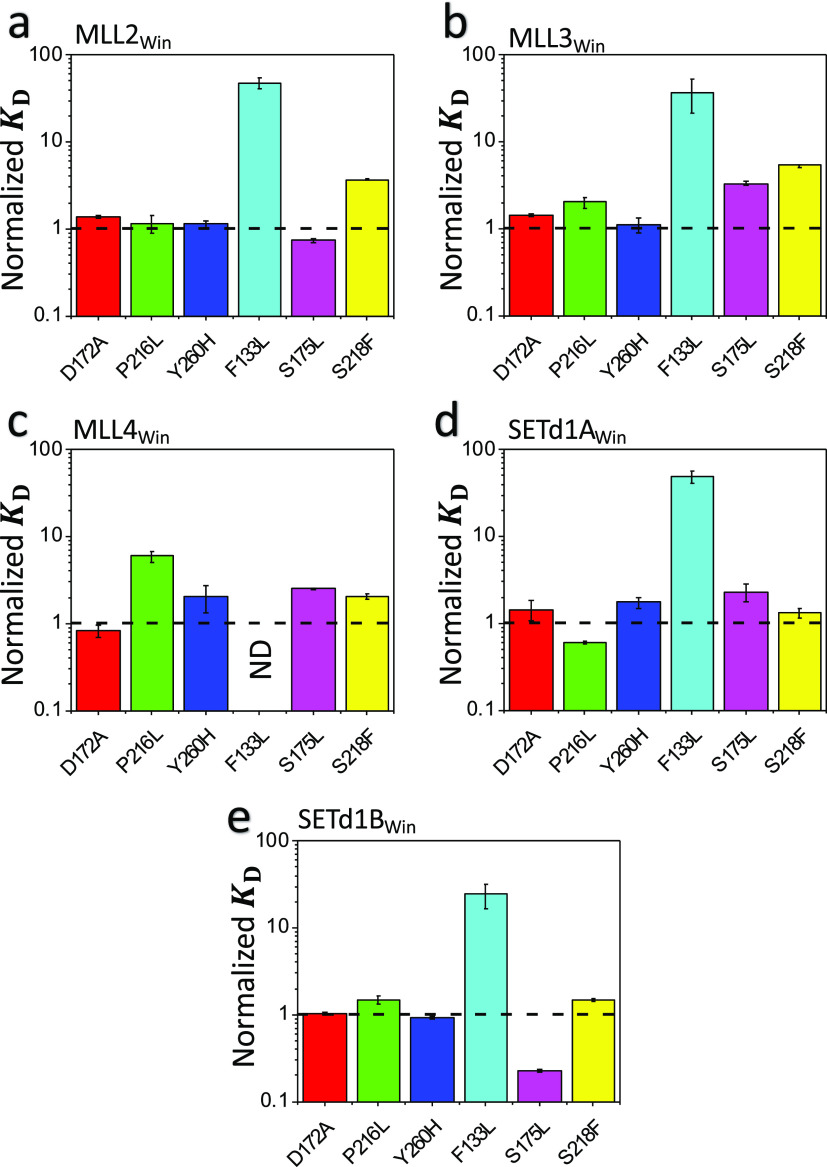
Normalized *K*_D_ of the WDR5 mutant-SET1_Win_ interactions using BLI sensorgrams. The *K*_D_ values for each SET1_Win_ ligand’s interaction with WDR5 mutants have been divided by the *K*_D_ of that SET1_Win_ ligand’s interaction with the native WDR5 protein. (a) MLL2_Win_, (b) MLL3_Win_, (c) MLL4_Win_, (d) SETd1A_Win_, and (e) SETd1B_Win_. ND stands for “Not Determined”. Using a BLI measurement, the interaction between F133L and MLL4_Win_ was detectable, but not quantifiable.

We then looked at surface mutants and the effects of these mutations on the WDR5-SET1_Win_ interaction. The normalized values of *k*_on_ (Figure S4), *k*_off_ (Figure 3), and dissociation constant *K*_D-BLI_ (Figure 4) for these WDR5 mutants are the values of these parameters of the SET1_Win_-WDR5 mutant pair interactions divided by those values corresponding to the SET1_Win_-native WDR5 pair interactions. In general, surface mutants D172A, P216L, and Y260H exhibited closely similar values of *k*_on_, *k*_off_, and *K*_D-BLI_ to those obtained for the native WDR5 protein (Tables S7–S9).^44^ Again, we were not able to obtain a quantifiable *k*_on_ for the MLL1_Win_-WDR5 mutant pair interactions due to limited time resolution of BLI. Interestingly, *k*_on_ followed the same trend with respect to SET1_Win_ peptides, as established in our previous study,^44^ with the lowest values for the neutrally charged MLL4_Win_, the highest values for the acidic SETd1A_Win_ and SETd1B_Win_, and the intermediate values for the positively charged MLL2_Win_ and MLL3_Win_. For example, for P216L-MLL4_Win_ interactions, *k*_on_ was (1.9 ± 0.3) × 10^4^ M^–1^ s^–1^. Yet, for the interactions of P216 L with MLL2_Win_, MLL3_Win_, SETd1A_Win_, and SETd1B_Win_*k*_on_ were (5.6 ± 0.8) × 10^4^ M^–1^ s^–1^, (5.3 ± 0.7) × 10^4^ M^–1^ s^–1^, (8.6 ± 0.8) × 10^4^ M^–1^ s^–1^, and (8.0 ± 0.8) × 10^4^ M^–1^ s^–1^, respectively. We conclude that *k*_on_ (0) < *k*_on_ (+1) < *k*_on_ (−1) for surface mutants, where the number between parentheses is the overall charge of the SET1_Win_ peptides (Table S1). In other words, *k*_on_ (MLL1_Win_, MLL4_Win_) < *k*_on_ (MLL2_Win_, MLL3_Win_) < *k*_on_ (SETd1A_Win_, SETd1B_Win_) for surface mutants. This *k*_on_ rule is likely determined by an asymmetric charge distribution in SET1_Win_ with respect to the highly conserved 6-residue Win motif peptide segment (P_–3_ through P_2_). Specifically, this is because of a positive charge located on the C-terminal flanking side in P_4_ (MLL2_Win_ and MLL3_Win_) and a negative charge located on the N-terminal flanking side in P_–7_ (SETd1A_Win_ and SETd1B_Win_). Asp-172 is located within the A pocket of WDR5 (Figure S2). SET1_Win_ ligands show no difference in their interactions with D172A as compared to the native WDR5 protein. We did not see any significant changes in the *k*_on_ and *k*_off_ for this pocket mutant. In addition, we noted a significantly weakened interaction of P216L with MLL4_Win_. Pro-216 is located within the B pocket.

### Cavity Mutants

In addition to D92N, we examined three WDR5 mutations within the WDR5 cavity, such as F133L, S175L, and S218F (Figure 2b). It has been previously reported that F133A significantly deteriorates the strength of the interactions of the MLL1 subunit with the WDR5-RbBP5-Ash2L subcomplex in vitro.^23^ Phe-133 is a critical neighboring WDR5 residue of the evolutionarily conserved Arg at P_0_ of SET1_Win_ ligands, contributing to a potentially strong cation−π interaction. Very weak interactions of F133L with MLL1_Win_ and MLL4_Win_ were not quantifiable using BLI. Here, F133L showed a decreased normalized *k*_on_ with MLL2_Win_, MLL3_Win_, SETd1A_Win_, and SETd1B_Win_ (Figure S4). As expected, F133L exhibited a noteworthy change in the *k*_off_ with respect to the native WDR5 protein (Figure 3), leading to a significant increase in the *K*_D-BLI_. For MLL2_Win_, MLL3_Win_, SETd1A_Win_, and SETd1B_Win_, these increased values spanned a range between 1 and 2 orders of magnitude (Figure 4; Table S9). This outcome indirectly confirms their close similarity in sequence and interaction mechanisms with WDR5.^35,36^ Their distinctions in binding affinities with respect to the other SET1_Win_ ligands can be attributed to the interaction of their flanking sides with the WDR5 surface.

However, the most interesting mutational effect is that of S175L, which has a more divergent impact on interactions of the SET1_Win_ peptides with respect to the native WDR5 protein. For example, S175L selectively weakens the interactions with MLL3_Win_, MLL4_Win_, and SETd1A_Win_, while substantially strengthening the interactions with SETd1B_Win_ (Figures 3 and 4; Table S9). Moreover, this change is primarily associated with a change in the *k*_off_. Ser-175 is part of a cluster of neighboring residues that co-participate in an array of hydrogen bonds, π–π, cation−π, and hydrophobic interactions with the conserved Arg in P_0_. These include Ser-91, Phe-133, Ser-175, Ser-218, Cys-261, Phe-263, and Ile-305.^35^ For example, Arg at P_0_ makes a water-mediated hydrogen bond with the Ser-175 backbone carbonyl group.^36^

Known SETd1B-WDR5 crystal structures suggest that replacing Ser-175 with Leu creates steric clashes that affect the structure of the B pocket (Figure S5).^35,36^ Specifically, it could displace Tyr-191 and make the pocket more hydrophobic, which would explain the increased affinity with SETd1B. It is worth mentioning that SETd1B is unique because it has a Phe residue at P_4_ (Table S1) that inserts into the hydrophobic B-pocket, while the other B-pocket binders have a more polar residue (Lys or Tyr) in that position. Interestingly, the increased affinity is made possible through a 4-fold decrease in the dissociation rate constant with no change in the association rate constant. Given the importance of slow dissociation rates for effective therapeutics,^50^ we predict molecules designed to take advantage of this interaction will improve dwell times and make more effective inhibitors.^21^

In agreement with prior crystallographic studies,^35,36^ S218F exhibited weakened interactions with MLL2_Win_, MLL3_Win_, and MLL4_Win_. However, its interactions with SETd1A_Win_ and SETd1B_Win_ were closely similar to those with the native WDR5 protein (Figures 3 and 4; Table S9). This finding is in accordance with a different mechanism of binding interactions of SETd1A_Win_ and SETd1B_Win_ with respect to the other SET1_Win_ ligands, likely due to an intermediate orientation of the C-terminal ends of SETd1A_Win_ and SETd1B_Win_ on the surface of blades 4 and 5.

### Validations of BLI Data and Qualitative Comparisons between Competing Techniques

**Figure 5:**
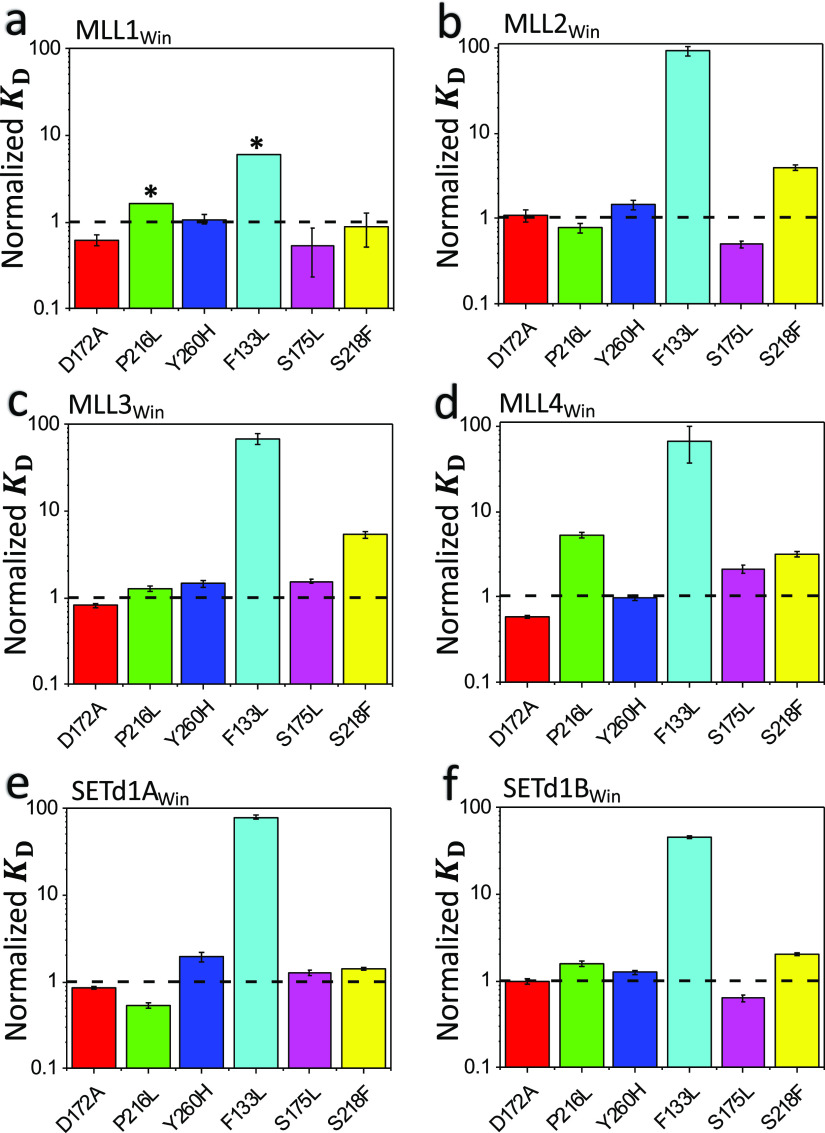
Normalized *K*_D_ of the WDR5 mutant-SET1_Win_ interactions using steady-state FP spectroscopy. The *K*_D_ values for each SET1_Win_ ligand’s interaction with WDR5 mutants have been divided by the *K*_D_ of that SET1_Win_ ligand’s interaction with the native WDR5 protein. (a) MLL1_Win_, (b) MLL2_Win_, (c) MLL3_Win_, (d) MLL4_Win_, (e) SETd1A_Win_, and (f) SETd1B_Win_. For vertical bars marked by “*”, the *K*_D_ of those interactions could not be determined. Those values represent the lower-limit of the *K*_D_ based on the highest WDR5 mutant concentrations used in this study.

To validate the outcomes of BLI measurements, we next used steady-state FP spectroscopy as an orthogonal technique (Figure S6) to determine binding affinities, *K*_D-FP_, of the interactions of SET1_Win_ ligands with WDR5 mutants (Figure 5; Table S10). 14-residue SET1_Win_ peptide ligands were fluorescently labeled with Sulforhodamine B at the N terminus and amidated at the C terminus. A 3 nm long Gly/Ser-rich peptide spacer was inserted between the fluorophore site and the SET1Win sequence. Then, steady-state FP anisotropy, *r*, values were collected at increasing WDR5 concentrations. Dose-response FP measurements enabled determinations of the *K*D-FP. Remarkably, the FP experiments validate all qualitative findings using BLI. These include the confirmation of very weak binding interactions of D92N with all SET1_Win_ ligands (Figure S7). In addition, we always found that the absolute *K*D values (i.e., not normalized) obeyed the following inequality: *K*D-BLI > *K*D-FP. This outcome confirms our previous results, indicating that measured interactions are stronger in unrestricted conditions than those corresponding to restrained conditions (Table S11).^44^ Because these WDR5 mutants have been examined using BLI and FP, we can also compare these approaches qualitatively.^65^ For example, using BLI, we can determine the kinetic fingerprint of these interactions. Yet, this cannot be inferred using steady-state FP spectroscopy. BLI is an immobilization-based technique, whereas FP is a method that probes the binding affinity in solution under unrestricted conditions. This is likely the reason why the *K*D-BLI is always about 1 order of magnitude greater than the *K*D-FP (Tables S9 and S10). In addition, FP measurements enabled us to measure some weaker interactions, which had kinetics that were too fast for the BLI time resolution (e.g., for MLL1_Win_). Furthermore, these approaches probe distinctive physical processes. On one hand, BLI is a real-time technique that samples both the association and dissociation phases based on alterations in the interference pattern of white light reflected on the sensor surface. On the other hand, steady-state FP is a time-independent technique that monitors changes in the rotational diffusion of a fluorescently labeled molecule upon its binding to another molecule.

**Figure 6:**
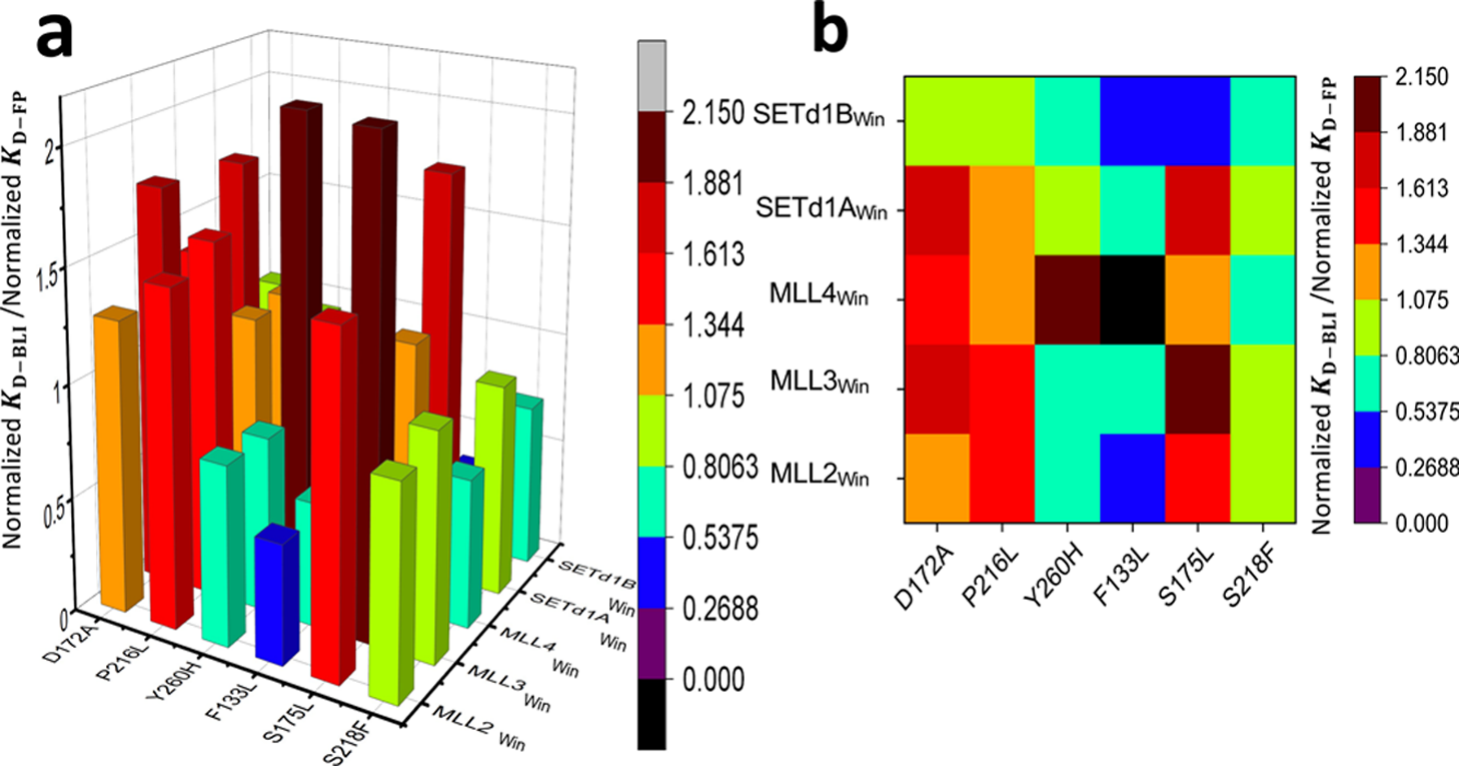
Quantitative comparison between affinity data resulting from BLI and FP measurements. (a) 3D graph of the ratio of the normalized *K*_D-BLI_ to the normalized *K*_D-FP_. (b) Two-dimensional heat map of the ratio of the normalized *K*_D-BLI_ to the normalized *K*_D-FP_. Normalized *K*_D_ values are the *K*_D_ measured for a specific WDR5 mutant-SET1_Win_ interaction pair divided by the *K*_D_ value corresponding to the native WDR5 protein.

We then calculated the ratio *K*_D-BLI_/*K*_D-FP_ (Figure S8). The variability of the *K*_D-BLI_/*K*_D-FP_ ratio for different interacting pairs was likely caused by two determinants: (i) the difference in mobility of each SET1_Win_ ligand with respect to WDR5 mutants, and (ii) the distinction in the physical processes probed by the two methods. To cancel the effect of these two determinants, we calculated another dimensionless parameter, the ratio of normalized *K*_D-BLI_/normalized *K*_D-FP_, which spanned a much narrower spectrum, between 0.36 and 2.15 (Figure 6; Table S12). This finding illuminates the qualitative agreement of data resulting from BLI and FP measurements, fortifying our conclusions on the effect of introducing these missense mutations on SET1_Win_-WDR5 interactions. Moreover, these BLI and FP data are in accordance with a recent single-molecule study using an engineered protein nanopore,^45^ which indicated unaffected D172A-MLL4_Win_ interactions and weak D92N-MLL4_Win_ interactions with respect to those of the native WDR5 protein. Taken together, these findings demonstrate the critical role of a negative charge located within the acidic WDR5 cavity for the strength of WDR5-SET1_Win_ interactions.

### Implications of Win Binding Site Mutants

Alterations in MLL/SET1 family enzymes are associated with genome-wide aberrations in the patterns of H3K4 methylation, which are linked to abnormal transcriptional programs that promote malignancy.^66−69^ WDR5 is a key component of MLL/SET1 family complexes and mutations in the Win binding site disrupt MLL complex assembly and enzymatic activity.^22,23,35,70^ Knockdown of WDR5 also alters global H3K4 methylation patterns resulting in developmental defects in vertebrates.^71^ Recent exome sequencing projects have uncovered numerous missense mutations in WDR5 and other subunits of MLL/SET1 family complexes, suggesting that loss-of-function may underly malignancy. Cell- and animal-based studies are needed to determine whether these mutations result in disease. Yet, prioritizing such costly experiments would benefit greatly from reliable preliminary information that is beyond the capabilities of most current computational methods.^72^ Thus, experimental studies to delineate how cancer-associated mutations impact the biochemical function of WDR5 remain an attractive approach to prioritize mutations for further study.

While the impact of these WDR5 mutations on WDR5-SET1_Win_ interactions is readily distinguishable, their effect on the overall assembly of the SET1 complexes and their functional features is a bit more nuanced. Given our understanding of SET1 family complex behavior,^16,48^ we can say that these inspected WDR5 mutations have a divergent impact. The absence of WDR5^16^ and/or the inhibition of SET1_Win_-WDR5 interactions^48^ downregulates the H3K4 di-methylation function of MLL1 and SETd1A, while this upregulates the H3K4 mono-methylation function of MLL3.^16,70^ Therefore, mutations that significantly disrupt SET1_Win_-WDR5 interactions are likely to have similar effects. Consequently, F133L and D92N should disrupt the di-methylation by MLL1 and SETd1A. Furthermore, the mono-methylation of MLL3 would be upregulated in the case of F133L, S218F, S175L, and D92N. Our results show that even within the Win binding site, given their effect on SET1_win_-WDR5 interactions, cavity mutations are more likely to be driver mutations instead of passenger mutations. This holds especially true for D92N, F133L, and S175L. For example, Ali and co-workers (2014) found that F133L disrupts the mitotic progression in the cell cycle process.^73^

Information that concerns the *K*_D_ values of mutations within the B pocket is critical for future drug development. Precision medicine depends on understanding the unique biophysical impacts of each missense mutation on the structure and function of putative oncogene proteins. Our data suggest that this knowledge would help researchers in deciding which inhibitors to use as potential therapeutic approaches. For example, this work indicates that individuals harboring a breast cancer S175L mutation in WDR5 are more likely to respond to inhibitors targeting the hydrophobic interactions in the B pocket than other inhibitors. Furthermore, the unique impact of S175L in SETd1B also implies that those cancers are due to perturbations in the SETd1B-catalyzed H3K4 methylation pathway. This type of information would greatly enhance our ability to prioritize cellular and animal-based follow-up studies that can address more specific hypotheses.

### Concluding Remarks

In this study, we evaluated key somatic cancer mutations of WDR5. Specifically, we used the CLUMPS approach to identify that these mutations accumulate within the Win binding site and extracted a representative subset of WDR5 mutants for determining the real-time kinetics of their interactions using high-throughput biosensing techniques. Our work shows that the total number of mutations in a tumor sample can be used as a parameter to filter out mutations. Furthermore, we noted that the Win site shows a substantial presence of low-*N* mutations, while the WBM site shows none. This helped us to exclusively focus on Win binding site mutants for further biophysical measurements. Therefore, we explored the effect of mutations in this binding site by evaluating a detailed kinetic fingerprint of corresponding interactions with various SET1_Win_ ligands. We provide experimental evidence for influential roles of the residues within the WDR5 cavity on the strength of these interactions. Steady-state FP spectroscopy measurements also confirmed outcomes resulting from BLI experiments. Finally, the interactions of WDR5 cavity mutants depended on the nature of the SET1_Win_ ligands. These divergent effects have distinctive impacts on H3K4 methylation, and therefore for the downstream expression of genes. This is a finding that can impact future strategies for the design, development, and optimization of inhibitors that are aimed at targeting the multitasking high-affinity Win binding site under oncogenic conditions. In the future, it would be interesting to examine the effect of the Win binding site cancer mutations on the kinetics and strength of the interactions of WDR5 with other protein partners and Win motif ligands.

## Materials and Methods

### Clustering of Mutations in Protein Structures

This approach was used as previously reported.^34^ WDR5 mutations were obtained using the COSMIC database^29,30,63^ and available X-ray crystallographic information (PDB code 4ERY).^35^ A WAP score was generated for the distribution of mutations using the following equation
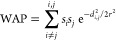
1where *i* and *j* iterated over all residues of WDR5. Here, *d*_*i*,*j*_ is the Euclidean distance between residues *i* and *j* in Angstroms, and *r* denotes the distance threshold set to a constant value of 6 Å.^34^*s*_i_ represents the normalized number of samples, in which the residue *i* was mutated. This parameter is given by
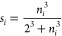
2where *n*_*i*_ represents the number of samples, in which the residue *i* was mutated. The *P*-value was determined by calculating the WAP score for 10^6^ random distributions of mutations, and then by comparing it with that value of the known distribution.

### Protein Expression and Purification

All expression plasmids were synthesized, codon optimized, and sequence verified by GenScript. Human WDR5 (UniProtKB—P61964; WDR5_HUMAN) and its mutants were expressed and purified as described previously.^22,23,44^

### Peptide Synthesis, Labeling, Purification, and Analysis

For BLI measurements, 14-residue SET1_Win_ peptide ligands were biotinylated at their N terminus and amidated at their C terminus. They were synthesized and purified to ≥95% purity by GenScript. Purity confirmation, amino acid analysis, and solubility testing were conducted and provided by GenScript. For steady-state FP measurements, details on peptide synthesis, labeling, purification, and analysis were previously published.^44^ In brief, peptides were synthesized using a Biotage Syro I peptide synthesizer (Biotage). Then, the peptides were purified using reversed-phase chromatography in two steps: (i) flash chromatography using a Biotage Isolera One (Biotage), and (ii) semi-preparative HPLC using a Waters 2695 separations module equipped with a Waters 2996 photodiode array detector. Fluorophore-containing peptide fractions were analyzed by matrix-assisted laser desorption ionization time-of-flight mass spectroscopy for the identity and purity tests.

### Biolayer Interferometry

Octet RED384 (FortéBio) was employed for the BLI studies.^44,58−60^ The assay buffer contained 150 mM NaCl, 20 mM Tris–HCl, 1 mM TCEP, 1 mg/mL bovine serum albumin, pH 7.5. Streptavidin-coated biosensors were incubated for 15 min with 5 nM biotin-tagged SET1_Win_ peptide to specifically immobilize an optimal level of peptide. Sensors were then rinsed briefly in an assay buffer to remove unbound peptides. Next, sensors were exposed to threefold serial dilutions of WDR5 for the association process. The dissociation phase was initiated by transferring the BLI sensors into WDR5-free buffer. For all WDR5 concentrations, binding curves were recorded by subtracting the baseline and the drift in the sensorgrams acquired with unloaded sensors. These BLI measurements were conducted at 24 °C. For various WDR5 concentrations, [*C*], the association phases were fitted using the following equation^74^
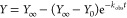
3where *Y*_0_ and *Y*_∞_ are the response signals at the initial time and infinity, respectively. *t* is the cumulative time of the association phase, whereas *k*_obs_ denotes the apparent first-order reaction rate constant of the association phase. The dissociation phases were fitted using the following equation
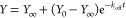
4where *Y*_0_ and *Y*_∞_ denote the responses at the initial time and infinity, respectively. *k*_off_ is the dissociation rate constant. The association rate constant, *k*_on_, was determined using the slope of the linear curve^62,75^
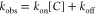
5

Using several WDR5 concentrations, we also conducted global fittings, which provided the corresponding *k*_on_ and *k*_off_ values. The equilibrium dissociation constants, *K*_D_, were indirectly determined using the *k*_on_ and *k*_off_ values (*K*_D_ = *k*_off_/*k*_on_). In each case, three independent BLI recordings were acquired for further determinations of the kinetics and dynamics of WDR5-SET1Win interactions.

### Steady-State Fluorescence Polarization (Steady-State FP) Measurements

Steady-state FP recordings were performed using a SpectraMax i3x plate reader (Molecular Devices).^61,76^ All steady-state FP measurements were conducted using a buffer that contained 20 mM Tris–HCl (pH 7.5), 150 mM NaCl, 1 mM TCEP, 0.005% Tween 20, and 96-well black untreated polystyrene microplates (Corning Inc). Other details of steady-state FP measurements were previously reported.^44^ 100 μL of each 20 nM labeled SET1_Win_ peptide was added to individual wells at a final concentration of 10 nM. The steady-state FP anisotropy was measured on the plates after a 1 h incubation at room temperature in the dark. WDR5-dependent dose-response data were averaged and then fitted using a four-parameter logistic function to acquire the binding affinity (*K*_D_) for each interaction pairs.

### Molecular Graphics

In this study, molecular graphics was conducted using the PyMOL Molecular Graphics System (Version 2.4.0 Schrödinger, LLC).

## Supporting Information Available

Sequence of SET1_Win_ peptide ligands; location of somatic cancer mutations from tumor samples with *N* < 500; results of mutation clustering for different mutation subsets; list of tumor locations associated with missense WDR5 mutations within and around the Win binding site; list of noncovalent bonds at the WDR5-SET1_Win_ interface; location of surface WDR5 mutations within the A and B pockets; BLI measurements of very weak interactions; kinetic rate constants of association and dissociation of WDR5 mutants with SET1_Win_ ligands; equilibrium dissociation constants of WDR5 mutants with SET1_Win_ ligands; structural information on the effect of the S175L mutation; steady-state FP spectroscopy curves for the interactions of WDR5 mutants with SET1_win_ ligands; equilibrium dissociation constants of WDR5 mutants with SET1_Win_ ligands using steady-state FP spectroscopy; quantitative comparisons of affinity data acquired with BLI and FP; and supporting references (PDF)
