# Supplementary material for: Convergent Alterations of a Protein Hub Produce Divergent Effects within a Binding Site
Source: ACS Chem Biol. 2022 May 25;17(6):1586–97. doi: 10.1021/acschembio.2c00273 (PMC9207812; doi:10.1021/acschembio.2c00273)
Supplement: Supplementary file 1 — cb2c00273_si_001.pdf [file cb2c00273_si_001.pdf]

SUPPORTING INFORMATION

Convergent Alterations of a Protein Hub Produce Divergent Effects Within a Binding Site

Ali Imran,[1] Brandon S. Moyer,[2] Dan Kalina,[2,3] Thomas M. Duncan,[4] Kelsey J. Moody,[1,2,3,5] Aaron J. Wolfe,[1,2,3,5] Michael S. Cosgrove,[4,*] and Liviu Movileanu[1,6,7,*]

[1]*Department of Physics, Syracuse University, 201 Physics Building, Syracuse, New York 13244-1130, USA*

[2]*Ichor Life Sciences, Inc., 2651 US Route 11, LaFayette, New York 13084, USA*

[3]*Department of Chemistry, State University of New York College of Environmental Science and Forestry, 1 Forestry Dr., Syracuse, New York 13210, USA*

[4]*Department of Biochemistry and Molecular Biology, State University of New York Upstate Medical University, 4249 Weiskotten Hall, 766 Irving Avenue, Syracuse, New York 13210, USA*

[5]*Lewis School of Health Sciences, Clarkson University, 8 Clarkson Avenue, Potsdam, New York 13699, USA*

[6]*Department of Biomedical and Chemical Engineering, Syracuse University, 329 Link Hall, Syracuse, New York 13244, USA*

[7]*The BioInspired Institute, Syracuse University, Syracuse, New York, 13244, USA*

Keywords: Mixed Lineage Leukemia (MLL); SET1 proteins; Protein-protein Interactions; Biolayer Interferometry; Fluorescence polarization; Protein engineering; Cancer; Bioinformatics.

*The corresponding authors' contact information:
Liviu Movileanu, PhD, E-mail: lmovilea@syr.edu
Michael Cosgrove, PhD, E-mail: cosgrovm@upstate.edu

CONTENTS OF THE SUPPORTING INFORMATION FILE

1. Sequence of 14-residue $SET1_{Win}$ peptide ligands (**Supplementary Table S1**).
2. Location of somatic cancer mutations from tumor samples with N < 500 and their distributions with a well-defined upper limit of mutations (**Supplementary Tables S2**-S3).
3. Results of mutation clustering for different N_{max}-based mutation subsets (**Supplementary Fig. S1**).
4. List of tumor locations associated with missense WDR5 mutations within and around the Win binding site (**Supplementary Table S4**)
5. List of noncovalent bonds at the WDR5-$SET1_{Win}$ protein interface (**Supplementary Tables S5**-S**6**).
6. Location of surface WDR5 mutations within the A and B pockets (**Supplementary Fig. S2**).
7. Very weak interactions of D92N with $SET1_{Win}$ ligands are detected by BLI measurements (**Supplementary Fig. S3**).
8. Kinetic rate constants of association of WDR5 mutants with $SET1_{Win}$ ligands using BLI measurements (**Supplementary Table S7**).
9. Kinetic rate constants of dissociation of WDR5 mutants with $SET1_{Win}$ ligands using BLI measurements (**Supplementary Table S8**).
10. Normalized kinetic rate constants of association of WDR5 mutants with $SET1_{Win}$ ligands using BLI measurements (**Supplementary Fig. S4**).
11. Equilibrium dissociation constants of WDR5 mutants with $SET1_{Win}$ ligands using BLI measurements (**Supplementary Table S9**).
12. Structural information on the effect of the S175L mutation (**Supplementary Fig. S5**).
13. Steady-state FP spectroscopy curves for the interactions of WDR5 mutants with $SET1_{win}$ ligands (**Supplementary Fig. S6**).
14. Very weak interactions of D92N with $SET1_{Win}$ ligands are detected by steady-state FP spectroscopy measurements (**Supplementary Fig. S7**).
15. Equilibrium dissociation constants of WDR5 mutants with $SET1_{Win}$ ligands using steady-state FP spectroscopy measurements (**Supplementary Table S10**).
16. Quantitative comparisons of affinity data acquired with BLI and FP (**Supplementary Fig. S8 Tables S11-S12)**.
17. Supporting references.

1. Sequence of 14-residue SET1$_{Win}$ peptide ligands

<u>Supplementary Table S1.</u> Amino acid sequences of SET1$_{Win}$ motif peptide ligands. Here, MLL1$_{Win}$, MLL2$_{Win}$, MLL3$_{Win}$, MLL4$_{Win}$, SETd1A$_{Win}$ and SETd1B$_{Win}$ are the following 14-residue SET1 Win motif ligands: MLL1$^{3758\text{-}3771}$, MLL2$^{5333\text{-}5346}$, MLL3$^{4703\text{-}4716}$, MLL4$^{2504\text{-}2517}$, SETd1A$^{1488\text{-}1501}$, and SETd1B$^{1698\text{-}1711}$.[1-3]

**SET1$_{Win}$**	P_{-7}	P_{-6}	P_{-5}	P_{-4}	P_{-3}	P_{-2}	P_{-1}	P_0	P_1	P_2	P_3	P_4	P_5	P_6	**Charge**
MLL1$_{Win}$	L	N	P	H	G	S	A	R	A	E	V	H	L	S*	0
MLL2$_{Win}$	I	N	P	T	G	C	A	R	S	E	P	K	I	L	+1
MLL3$_{Win}$	V	N	P	T	G	C	A	R	S	E	P	K	M	S	+1
MLL4$_{Win}$	L	N	P	H	G	A	A	R	A	E	V	Y	L	S**	0
SETd1A$_{Win}$	E	H	Q	T	G	S	A	R	S	E	G	Y	Y	P	-1
SETd1B$_{Win}$	E	H	V	T	G	C	A	R	S	E	G	F	Y	T	-1

S* This is an R3771S-substituted MLL1$_{Win}$ peptide ligand.
S** This is an R2517S-substituted MLL4$_{Win}$ peptide ligand.

2. Location of somatic cancer mutations from tumor samples with $N < 500$ and their distributions with a well-defined upper limit of mutations

<u>Supplementary Table S2.</u> Location of mutations from tumor samples with $N < 500$. The mutation distribution from tumor samples with low N values is shown below. Out of 8 such somatic cancer mutations, 5 were within or around the Win binding site, while 3 were found elsewhere. Mutated residues are either inside the WDR5 cavity (▪), have established interactions with SET1$_{Win}$ (▫), or sequentially are one residue away from residues with established interactions with SET1$_{Win}$ (◦).[1,4]

**Mutations within and around the Win binding site**	**Independent Mutations**
F133L ▪ ▫<br>S175L ▪ ▫<br>A264V ▪<br>N130Y ◦<br>D150G ◦	S54N<br>G254D<br>L282P

Supplementary Table S3. Distributions of somatic cancer mutations in tumors with a well-defined upper limit of mutations. Diverse mutation clusters correspond to different maximum number of mutations, N_{max}. The mutations highlighted in yellow were studied further using BLI and steady-state FP spectroscopy. S218F and D92N mutations, which are located within the WDR5 cavity, were found in tumors with $N > 10,000$. For compiling these mutations, the COSMIC database was used.[5-7]

Condition	Mutations
$N < 10,000$	N130Y S54N S175L G254D A264V D150G F133L L282P L185F R181C E292Q S184F D333Y S148F I327V S318P P311Q S223Y S202L D213N P139H Y75H V217M T253A L143F R181C Y260H P224S A201T D172A L102V R196C V275L G226V G299C D172N E279K L206P D302E Q289E S278L K245N H255N G277D I274F S91F S171L P216L L102F L230P G147E
$N < 5,000$	N130Y S54N S175L G254D A264V D150G F133L L282P L185F R181C E292Q S184F D333Y S148F I327V S318P P311Q S223Y S202L D213N P139H Y75H V217M T253A L143F R181C Y260H P224S A201T D172A L102V R196C V275L
$N < 1,000$	N130Y S54N S175L G254D A264V D150G F133L L282P L185F R181C E292Q
$N < 500$	N130Y S54N S175L G254D A264V D150G F133L L282P

3. Results of mutation clustering for different N_{max}-based mutation subsets

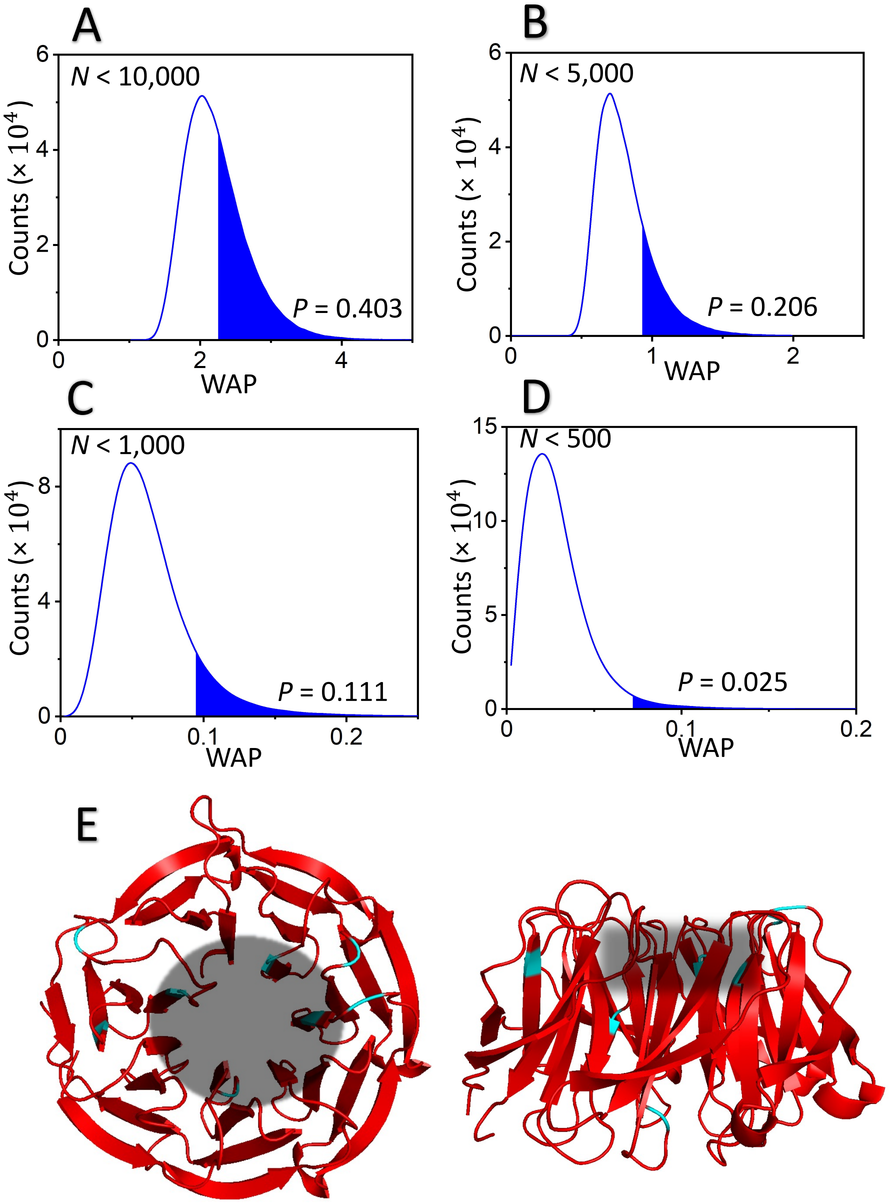

__Supplementary Fig. S1.__ ***Results of mutation clustering for different N_{max}-based mutation subsets.*** **(A)** WAP scores were calculated using 4 different subsets of mutations divided on the basis of the genetic damage, *N*, in their corresponding tumors. The *P*-values were calculated by comparing to the calculated WAP scores to those corresponding to random permutations of the mutation distribution. 10^6 configurations were used for $N < 10,000$ **(A)**, $N < 5,000$ **(B)**, $N < 1,000$ **(C)**, and $N < 500$ **(D)**. **(E)** The top and side views of WDR5 shown in red, on the left and right sides, respectively. The locations of low-*N* mutations are marked in cyan. The shaded region in dark grey represents the Win binding site of WDR5. These representations were made using pdb entry code 4ERY.[1]

4. List of tumor locations associated with missense WDR5 mutations within and around the Win binding site

<u>Supplementary Table S4.</u> Tumor locations associated with missense WDR5 mutations. The table shows the location of the tumors in which the mutations were found.

<table>
<tr><th>Mutation</th><th>Tumor Location</th><th>Other information</th></tr>
<tr><td>D172A***</td><td>Kidney</td><td rowspan="7">Studied experimentally by BLI and steady-state FP</td></tr>
<tr><td>P216L</td><td>Skin</td></tr>
<tr><td>Y260H</td><td>Large Intestine</td></tr>
<tr><td>S218F</td><td>Skin</td></tr>
<tr><td>D92N***</td><td>Endometrium</td></tr>
<tr><td>F133L*</td><td>Kidney</td></tr>
<tr><td>S175L*</td><td>Breast</td></tr>
<tr><td>A264V</td><td>Soft Tissue</td><td rowspan="6">Clustering with $N < 500$</td></tr>
<tr><td>N130Y</td><td>Breast</td></tr>
<tr><td>D150G</td><td>Stomach</td></tr>
<tr><td>S54N</td><td>Biliary Tract</td></tr>
<tr><td>G254D</td><td>Haematopoietic and Lymphoid Tissue</td></tr>
<tr><td>L282P</td><td>Kidney</td></tr>
<tr><td>S91F**</td><td>Skin</td><td>This mutant disrupts known Win binding site interactions</td></tr>
</table>

*F133L and S175L also meet the clustering with $N < 500$ condition. It was found that F133L disrupts the mitotic progression in the cell cycle process.[8]

S91F was not studied experimentally. It does not meet the clustering with the $N < 500$ condition, yet it can disrupt known Win site interactions (Supplementary Table S5**). For example, a related mutant, S91K, is not able to make interactions with a minimal C-terminal SET catalytic domain of MLL1.[9]

***D172A and D92N were experimentally studied using single-molecule electrical recordings and an $MLL4_{Win}$-containing engineered nanopore.[3] In addition, D172A was recently studied using pull-down assays, showing declined interactions with histone H3 peptides with respect to the native WDR5 protein.[10]

5. List of noncovalent bonds at the WDR5-SET1$_{Win}$ protein interface

<u>Supplementary Table S5.</u> Mapping of hydrogen bonds at the WDR5-SET1$_{Win}$ interface. These results were obtained using previously published co-crystallization data of Dharmarajan and co-workers.[1] The cut-off distance for identifying these hydrogen bonds was 4.0 Å. Here, BB and SC denote backbone and side chain, respectively. These interactions were determined using protein interactions calculator (PIC).[11] The structures were not always able to model the whole sequence of the peptides, so the list of these hydrogen bonds is not comprehensive. The first residue in each bond belongs to the SET1$_{Win}$ ligand, whereas the second one belongs to WDR5. Only peptide sequences of the segments that were able to model these interactions are listed below. Entries with multiple distances represent multiple different hydrogen bonds formed by the same residues.

Peptide	Hydrogen Bonds	Distance (Å)	Type
MLL1$_{Win}$	G3762-G89	3.0	BB-BB
LNPHGSARAEVHL	H3761-K46	3.3	BB-SC
	S3763-I90	3.2	BB-SC
	A3764-S91	3.3,	BB-SC
	A3764-D107	3.0, 3.3	BB-SC
	R3765-S91	3.0, 2.8	BB-SC
	R3765-F133	3.0	BB-SC
	R3765-C261	2.9	BB-SC
	H3761-D107	2.7	SC-SC
MLL2$_{Win}$	G5337 – G89	3.1	BB-BB
INPTGCARSEPKI	K5344 – K259	2.9	BB-BB
	C5338-I90	3.7	BB-SC
	A5339 – D107	2.9	BB-SC
	R5340 – S91	3.1, 2.8	BB-SC
	R5340 – F133	3.0	BB-SC
	R5340 – C261	2.9	BB-SC
	C5338-S91	3.7	SC-SC
	N5334-D107	2.8	SC-SC
MLL3$_{Win}$	G4707-G89	3.3	BB-BB
VNPTGCARSEPKMS	K4714 – K259	2.9	BB-BB
	C4708-I90	3.5	BB-SC
	A4709 – D107	2.9, 3.5	BB-SC
	R4710 – S91	3.1, 2.8	BB-SC
	R4710 – F133	2.9	BB-SC
	R4710 – C261	2.9	BB-SC
	C4708-S91	3.7	SC-SC
	N4704-D107	2.8	SC-SC
MLL4$_{Win}$	G2508-G89	3.3	BB-BB
LNPHGAARAEVY	A2510-S91	3.4	BB-SC
	A2510 – D107	3.0	BB-SC
	R2511 – S91	3.0, 2.8	BB-SC
	R2511 – F133	3.0	BB-SC

	R2511 – C261	2.9	BB-SC
	N2505-D107	2.7	SC-SC
	Y2515-D172	3.2	SC-SC
SETd1A$_{Win}$ QTGSARSEGY	G1492-G89	3.3	BB-BB
	Y1499 – K259	2.8	BB-BB
	T1491-G322	3.5	BB-SC
	A1494-S91	3.4	BB-SC
	A1494 – D107	3.0, 3.4	BB-SC
	R1495 – S91	3.0, 2.8	BB-SC
	R1495 – F133	3.1	BB-SC
	R1495 – C261	2.9	BB-SC
SETd1B$_{Win}$ GCARSEG	G1702-G89	3.3	BB-BB
	C1703-I90	3.4	BB-SC
	A1704-S91	3.5	BB-SC
	A1704 – D107	3.0, 3.3	BB-SC
	R1705 – S91	3.2, 2.8	BB-SC
	R1705 – F133	3.1	BB-SC
	R1705 – C261	2.8	BB-SC
	C1703-S91	3.7	SC-SC

<u>Supplementary Table S6.</u> List of all known noncovalent interactions. These results were obtained in a similar method as that for **Table S5**. For each interaction, the first residue corresponds to the SET1$_{Win}$ ligand, while the second residue corresponds to WDR5. The cut-off radii for the ionic and cation-pi interactions were 6 Å. Also, the cut-off radii for the hydrophobic and aromatic-aromatic interactions were 5 and 7 Å, respectively.

**Peptide**	**Ionic**	**Cation-Pi**	**Hydrophobic**	**Aromatic-Aromatic**
MLL1$_{Win}$<br>LNPHGSARAEVHL	R3765-D92<br>H3761-D107<br>H3769-D172	R3765-F133<br>R3765-F263	A3766-A47<br>A3766-A65<br>A3764-Y131<br>A3764-F133<br>A3764-D149<br>V3768-Y260<br>A3766-K321	
MLL2$_{Win}$<br>INPTGCARSEPKI	R5340-D92	R5340-F133<br>R5340-F263<br>K5344-Y191	A5339-Y131<br>A5339-F133<br>A5339-F149<br>P5343-Y260	
MLL3$_{Win}$<br>VNPTGCARSEPKMS	R4710-D92	R4710-F133<br>R4710-F263<br>K4714-Y191	A4709-Y131<br>A4709-F133<br>A4709-F149<br>P4713-Y260	
MLL4$_{Win}$<br>LNPHGAARAEVY	R2511-D92<br>H2507-Q322	R2511-F133<br>R2511-F263	A2512-A47<br>A2509-A65<br>A2512-A65<br>A2510-Y131<br>A2510-F133<br>A2510-F149<br>Y2515-F149<br>Y2515-P173<br>Y2515-Y191<br>V2514-Y260<br>A2512-L321	Y2515-F149<br>Y2515-Y191
SETd1A$_{Win}$<br>QTGSARSEGY	R1495-D92	R1495-F133<br>R1495-F263	A1494-Y131<br>A1494-F133<br>A1494-F149<br>Y1499-Y191<br>Y1499-P216<br>Y1499-L234	Y1499-Y191
SETd1B$_{Win}$<br>GCARSEG	R1705-D92	R1705-F133<br>R1705-F263	A1704-Y131<br>A1704-F133<br>A1704-F149	

6. Location of surface WDR5 mutations within the A and B pockets

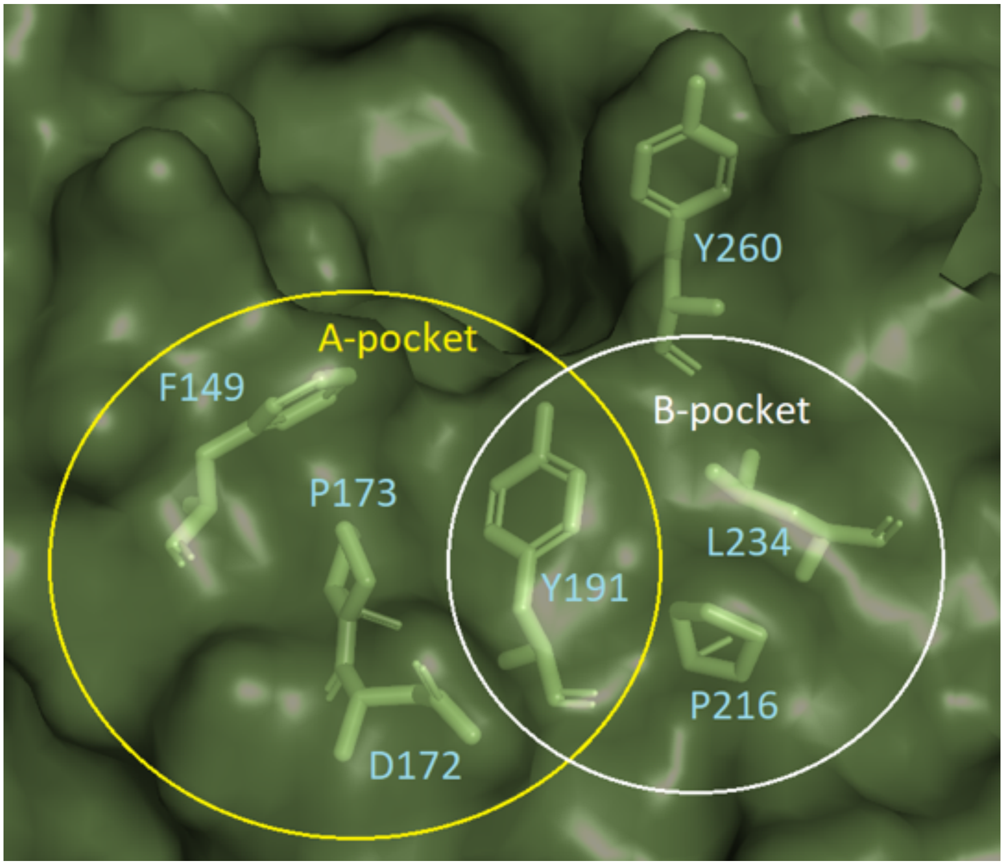

<u>Supplementary Fig. S2.</u> Cartoon illustrating the location of key residues present in the A and B pockets of the WDR5 protein.

7. Very weak interactions of D92N with SET1$_{Win}$ ligands are detected by BLI measurements

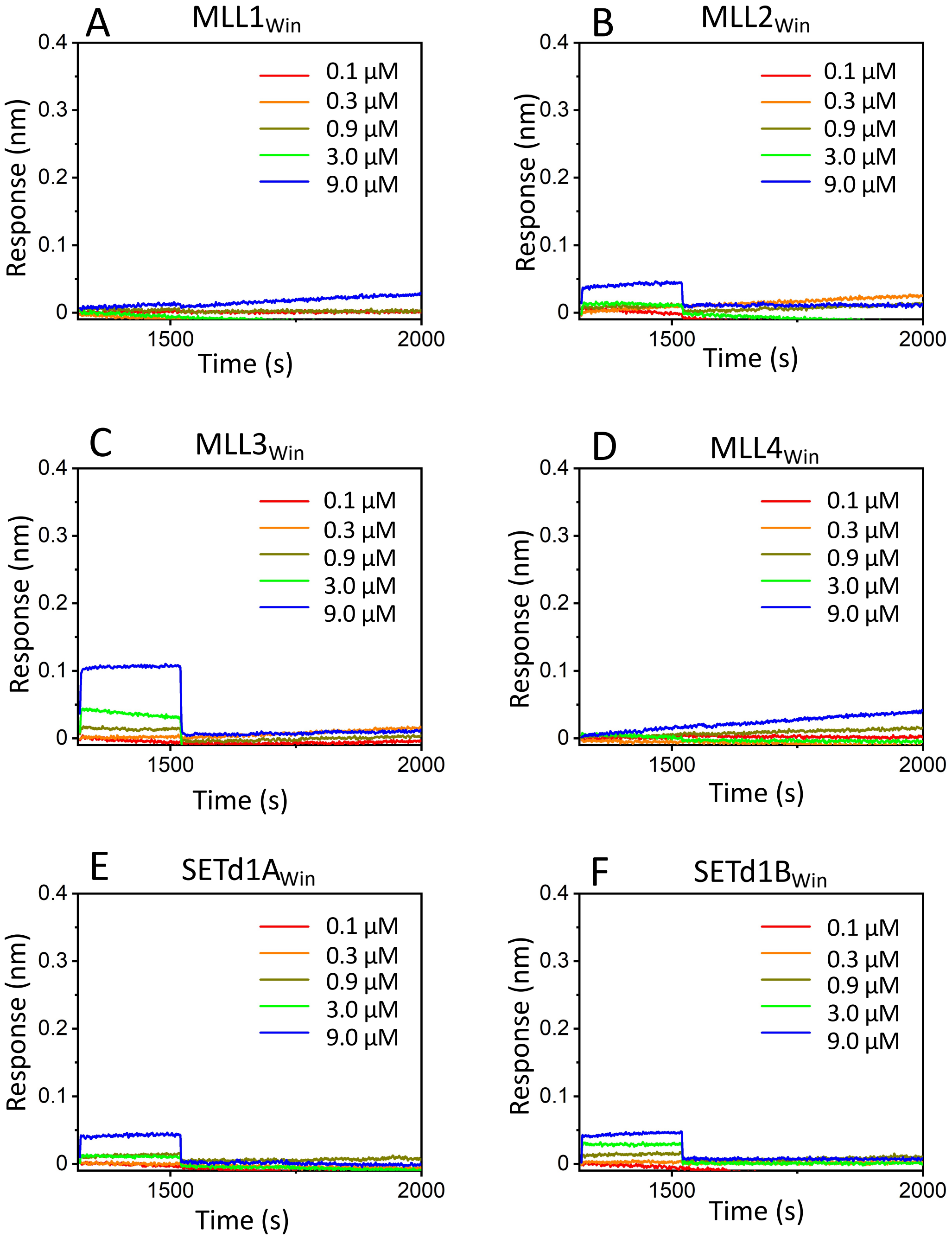

<u>Supplementary Fig. S3.</u> **BLI sensorgrams show either nondetectable or weakly detectable interactions of D92N with SET1$_{Win}$ ligands.** 5 nM biotin-tagged peptides were loaded onto streptavidin (SA) sensors for 15 minutes. A 3-fold serial dilution of D92N ranging from 0.1 μM to 9 μM was used to obtain individual binding curves. These sensorgrams show no binding interactions for MLL1$_{Win}$ and MLL4$_{Win}$. For the other SET1$_{Win}$ ligands, the binding interactions were visible, yet quantitative kinetic determinations were not possible. (**A**) MLL1$_{Win}$, (**B**) MLL2$_{Win}$, (**C**) MLL3$_{Win}$, (**D**) MLL4$_{Win}$, (**E**) SETd1A$_{Win}$, and (**F**) SETd1B$_{Win}$.

8. Kinetic rate constants of association of WDR5 mutants with SET1$_{Win}$ ligands using BLI measurements

Supplementary Table S7. Kinetic rate constants of association, k_{on}, of WDR5 mutants with the SETd1A$_{Win}$ ligands using BLI measurements. k_{on} values were provided in 10^4 $M^{-1}s^{-1}$. Results for WDR5 mutants were obtained the same way as those for the native WDR5 protein. For F133L, 3-fold serial dilutions ranging from 0.3 μM to 27 μM were used. Numbers represent mean ± s.d. determined from three independent experimental observations. D92N did not show any measurable binding interactions using BLI, so it was not included in this table.

Ligand	WDR5*	D172A	P216L	Y260H	F133L	S175L	S218F
MLL1$_{Win}$	~ 1**	~ 1**	~ 1**	~ 1**	NO***	~ 1**	~ 1**
MLL2$_{Win}$	4.4 ± 0.4	5.7 ± 0.6	5.6 ± 0.8	6.6 ± 0.9	1.6 ± 0.1	4.9 ± 0.4	4.0 ± 0.2
MLL3$_{Win}$	5.4 ± 0.6	6.5 ± 1.3	5.3 ± 0.7	6.4 ± 0.2	3.6 ± 2.1	4.9 ± 0.3	3.9 ± 0.5
MLL4$_{Win}$	2.3 ± 0.2	3.7 ± 0.4	1.9 ± 0.3	2.4 ± 0.3	~ 1	3.0 ± 0.1	2.0 ± 0.2
SETd1A$_{Win}$	8.2 ± 0.8	13 ± 1	8.6 ± 0.8	8.8 ± 1.3	2.6 ± 0.3	6.1 ± 0.7	5.6 ± 0.4
SETd1B$_{Win}$	7.1 ± 0.5	8.9 ± 1.4	8.0 ± 0.8	10 ± 1	4.3 ± 1.2	8.4 ± 0.6	6.3 ± 0.6

*This data are from Imran and co-workers (2021).[2]
**In this case, k_{on} was in the order of 10^4 $M^{-1}s^{-1}$ assuming that the association process is in the range of values determined with the other SET1$_{Win}$ ligands.
***NO stands for "Not Observed."

9. Kinetic rate constants of dissociation of WDR5 mutants with SET1$_{Win}$ ligands using BLI measurements.

Supplementary Table S8. Kinetic rate constants of dissociation, k_{off}, of WDR5 mutants with the SET1$_{Win}$ ligands using BLI measurements. k_{off} values were provided in 10^{-3} s^{-1}. Numbers represent mean ± s.d. determined from three independent experimental observations. D92N did not show any measurable binding interactions using BLI, so it was not included in this table.

Ligand	WDR5*	D172A	P216L	Y260H	F133L	S175L	S218F
MLL1$_{Win}$	> 1000**	> 1000**	> 1000**	> 1000**	NO***	> 1000**	> 1000**
MLL2$_{Win}$	7.7 ± 0.2	14 ± 1	11 ± 2	13 ± 1	140 ± 20	6.3 ± 0.1	25 ± 2
MLL3$_{Win}$	5.3 ± 0.1	9.3 ± 1.9	11 ± 3	7.7 ± 0.6	100 ± 10	16 ± 2	21 ± 3
MLL4$_{Win}$	39 ± 2	51 ± 10	220 ± 50	47 ± 28	> 1000*	130 ± 4	71 ± 1
SETd1A$_{Win}$	51 ± 6	110 ± 30	33 ± 4	98 ± 29	690 ± 40	89 ± 30	46 ± 7
SETd1B$_{Win}$	17 ± 1	23 ± 3	30 ± 5	23 ± 3	240 ± 20	4.9 ± 0.3	23 ± 2

*This data are from Imran and co-workers (2021).[2]
**This upper-limit value for the detection of k_{off} is set according to instrument specifications.
***NO stands for "Not Observed."

10. Normalized kinetic rate constants of association of WDR5 mutants with $SET1_{Win}$ ligands using BLI measurements

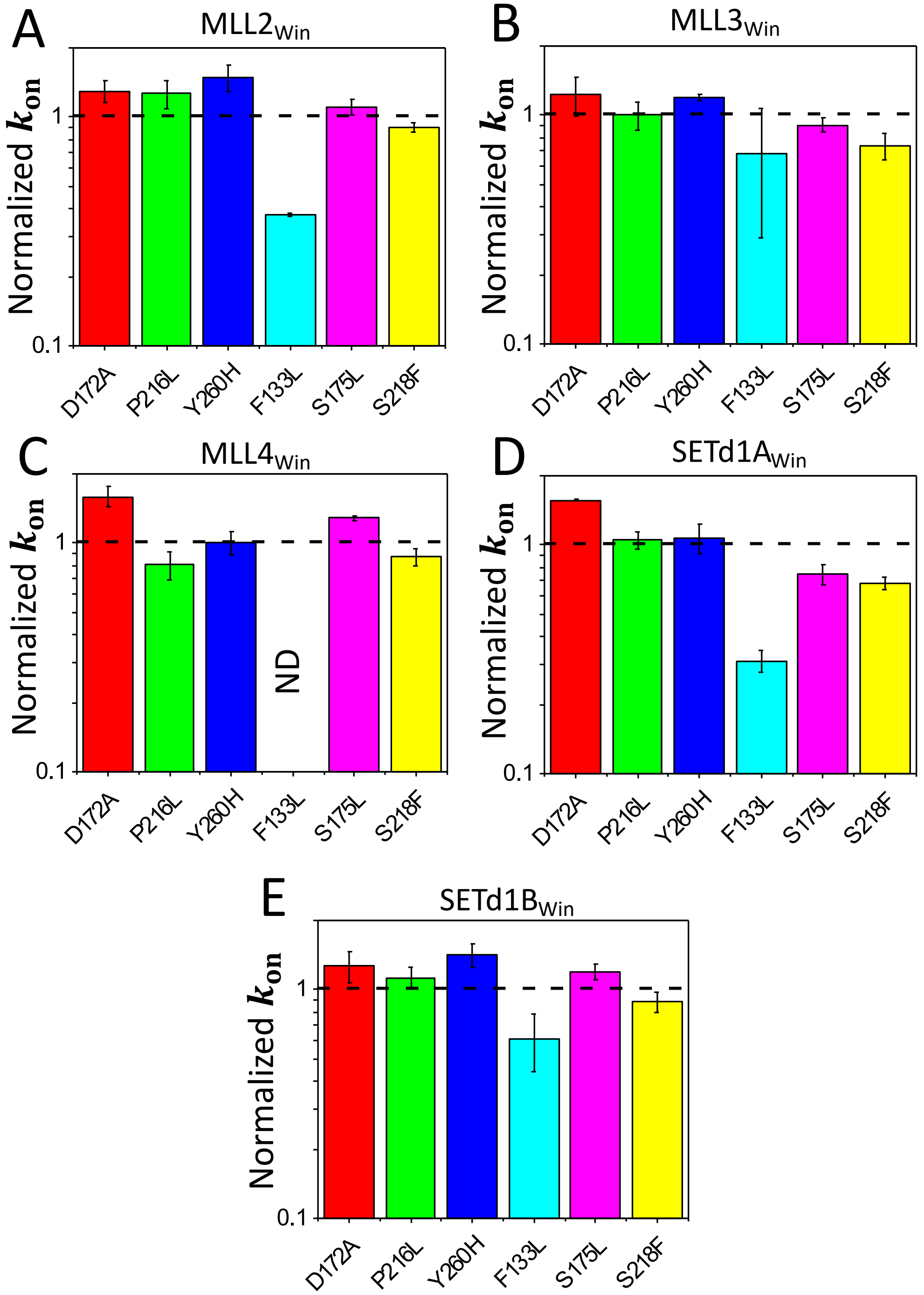

<u>Supplementary Fig. S4:</u> Normalized association rate constants of the WDR5-$SET1_{Win}$ interactions using BLI sensorgrams. The k_{on} values for each $SET1_{Win}$ ligand's interaction with WDR5 mutants have been divided by the k_{on} of that $SET1_{Win}$ ligand's interaction with the native WDR5 protein. ND stands for "Not Determined." Interaction between F133L and MLL4 was detectable, but not quantifiable, using a BLI measurement.

11. Equilibrium dissociation constants of WDR5 mutants with SET1$_{Win}$ ligands using BLI measurements.

Supplementary Table S9. Equilibrium dissociation constants, $K_{D\text{-}BLI}$, of the WDR5 mutants with the SET1$_{Win}$ ligands determined from BLI measurements. $K_{D\text{-}BLI}$ values are provided in nM. Numbers represent mean ± s.d. determined from three independent experimental determinations. D92N did not show any measurable binding interactions using the BLI, so it was not included in this table.

Ligand	WDR5#	D172A	P216L	Y260H	F133L	S175L	S218F
MLL1$_{Win}$	≳100,000*	≳ 100,000*	≳ 100,000*	≳ 100,000*	NO**	≳ 100,000*	≳ 100,000*
MLL2$_{Win}$	170 ± 20	240 ± 10	200 ± 50	200 ± 20	8,300 ± 1,000	130 ± 10	640 ± 20
MLL3$_{Win}$	100 ± 5	140 ± 10	200 ± 30	110 ± 20	3,700 ± 1,600	330 ± 20	530 ± 20
MLL4$_{Win}$	1,700 ± 200	1,400 ± 200	9,900 ± 1,400	3,400 ± 1,200	≳ 100,000*	4,200 ± 100	3,400 ± 300
SETd1A$_{Win}$	620 ± 20	900 ± 240	380 ± 20	1,100 ± 200	30,000 ± 5,000	1,400 ± 300	830 ± 110
SETd1B$_{Win}$	250 ± 30	260 ± 10	370 ± 40	230 ± 10	6,000 ± 1,900	56 ± 3	360 ± 20

#This data are from Imran and co-workers (2021).[2]

*This upper-limit value for the detection of $K_{D\text{-}BLI}$ results from dividing the upper-limit value of the detection of k_{off} by the value of the k_{on} approximation.

**NO stands for "Not Observed."

12. Structural information on the effect of the S175L mutation.

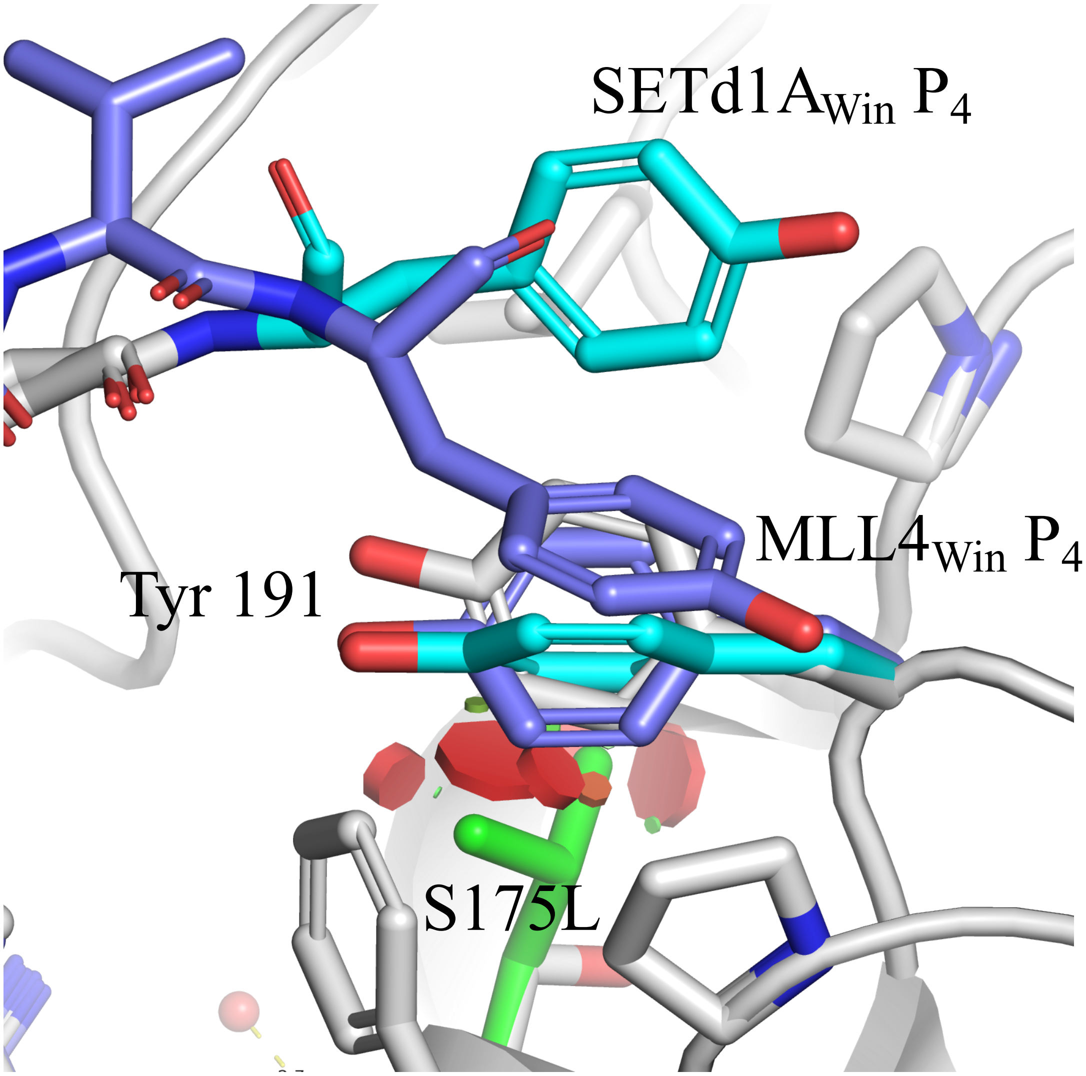

<u>Supplementary Fig. S5.</u> The effect of the S175L mutation on the SETd1A$_{Win}$-S175L interaction. The figure shows the effect of the S175L mutation on neighboring residues. It shows superimposed structures from PDB 4es0, 4ewr and 4erz. SETd1A$_{Win}$ is marked in cyan, while MLL4$_{Win}$ is marked in light blue. The red circles show the steric clashes created by replacing Ser-175 with Leu-175 (green). Superimposed Tyr-191 side chains from the three PDB files are shown. SETd1A$_{Win}$ was used, instead of SETd1B$_{Win}$, to show the B-pocket interactions, because the P$_6$ residue in the SETd1B$_{Win}$ structure is disordered.[1]

13. Steady-state FP spectroscopy curves for the interactions of WDR5 mutants with SET1$_{win}$ ligands

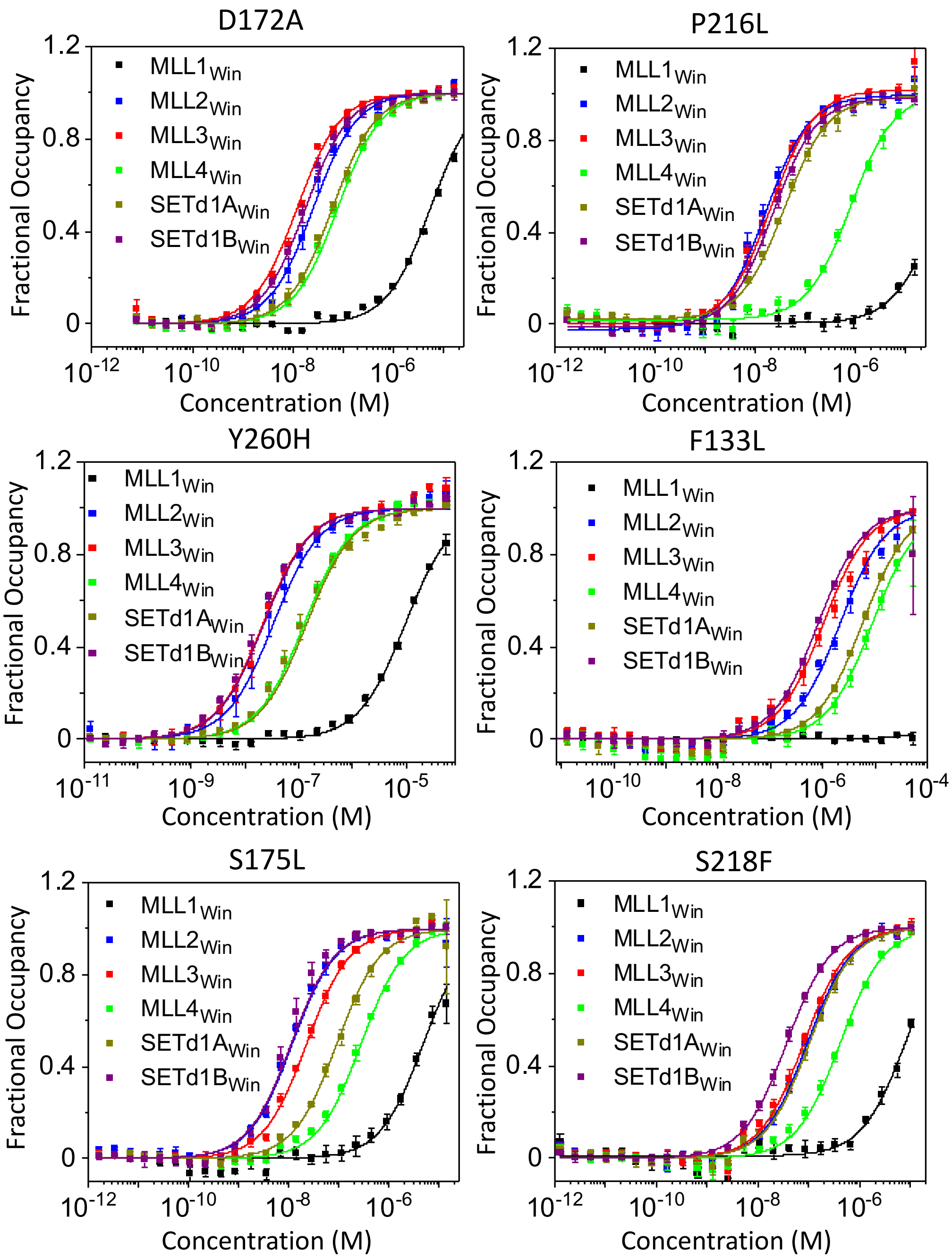

<u>Supplementary Fig. S6.</u> Steady-state FP anisotropy curves for WDR5-SET1$_{Win}$ ligand interactions. The N terminus of the SET1$_{Win}$ ligands was tagged with Sulforhodamine B, whereas the C terminus was amidated. The final concentration of the labeled SET1$_{Win}$ ligands in each well was 10 nM. Each SET1$_{Win}$ ligand – WDR5 run involved a 2-fold serial dilution of WDR5 over 24 wells. Three independent experiments were conducted to obtain the dose response, which was fitted using a four-parameter logistic model to get the K_D.

14. Very weak interactions of D92N with SET1$_{Win}$ ligands are detected by steady-state FP spectroscopy measurements

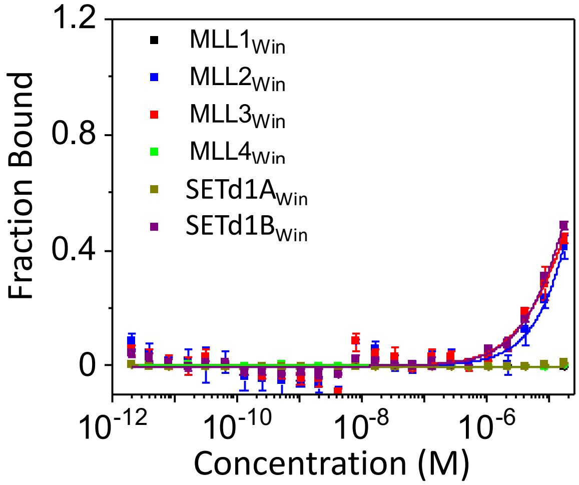

<u>Supplementary Fig. S7.</u> Fraction bound of SET1$_{Win}$ ligands to D92N using steady-state FP spectroscopy experiments. These experiments showed either nondetectable or very weak interactions of D92N with all SET1$_{Win}$ ligands. The final SET1$_{Win}$ ligand concentration was 10 nM. The WDR5 concentration (horizontal axis) spanned from low µM to low pM. No FP signal was detectable for MLL1$_{Win}$, MLL4$_{Win}$, and SETd1A$_{Win}$. Very weak interactions with D92N were detected in the case of MLL2$_{Win}$, MLL3$_{Win}$, and SETd1B$_{Win}$. For these SET1$_{Win}$ ligands, K_d was greater than 10 µM.

15. Equilibrium dissociation constants of WDR5 mutants with SET1$_{Win}$ ligands using FP measurements

<u>Supplementary Table S10.</u> Equilibrium dissociation constants, $K_{D\text{-}FP}$, of WDR5 mutants with SET1$_{Win}$ ligands determined from steady-state FP measurements. Three independent experiments were conducted to obtain the dose response, which was fitted using a four-parameter logistic model. $K_{D\text{-}FP}$ values are provided in nM. For D92N, the K_D values were greater than 17,000 nM. Numbers represent mean ± s.e.m. from three individual experimental determinations.

Ligand	WDR5#	D172A	P216L	Y260H	F133L	S175L	S218F
MLL1$_{Win}$	9,000 ± 5,500	5,600 ± 800	> 14,650*	9,700 ± 1,200	> 53,000*	4,800 ± 2,800	8,100 ± 3,400
MLL2$_{Win}$	23 ± 5	25 ± 4	18 ± 3	34 ± 4	2,100 ± 300	11 ± 1	94 ± 7
MLL3$_{Win}$	15 ± 4	12 ± 1	19 ± 1	22 ± 2	1,000 ± 100	23 ± 1	80 ± 7
MLL4$_{Win}$	130 ± 20	78 ± 3	690 ± 60	130 ± 10	8,800 ± 3,900	280 ± 30	420 ± 40
SETd1A$_{Win}$	72 ± 5	61 ± 2	39 ± 2	140 ± 20	5,700 ± 300	92 ± 7	100 ± 10
SETd1B$_{Win}$	18 ± 2	18 ± 1	28 ± 2	23 ± 2	820 ± 30	11 ± 1	37 ± 1

#These data are from the reference.[2]

*These low-value limits are based on the highest concentrations of WDR5 mutants employed in this study.

16. Quantitative comparisons of affinity data acquired with BLI and FP.

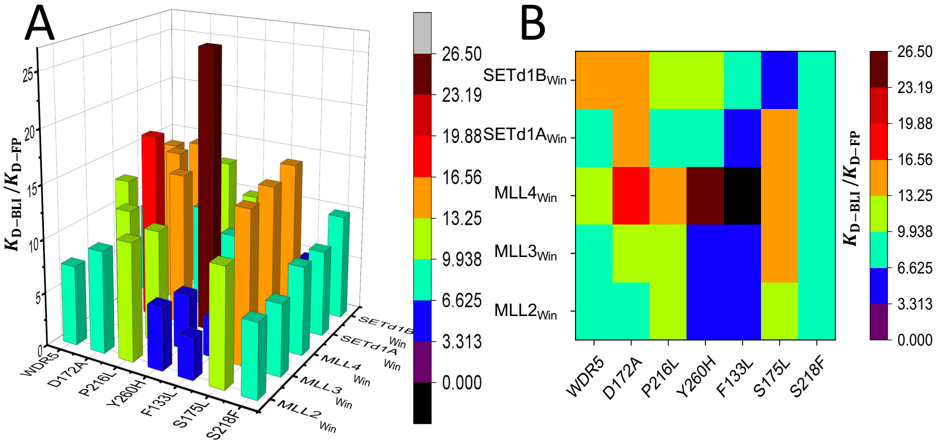

<u>Supplementary Fig. S8:</u> Quantitative comparison between affinity data resulting from BLI and FP measurements. (**A**) A 3D graph of the ratio of the $K_{D\text{-}BLI}$ to the $K_{D\text{-}FP}$. (**B**) A 2D heat map of the ratio of the $K_{D\text{-}BLI}$ to the $K_{D\text{-}FP}$.

<u>Supplementary Table S11.</u> Quantitative comparisons between affinity data resulting from BLI and FP measurements in terms of the ratio of the $K_{D\text{-}BLI}$ to the $K_{D\text{-}FP}$.

Protein	MLL2$_{Win}$	MLL3$_{Win}$	MLL4$_{Win}$	SETd1A$_{Win}$	SETd1B$_{Win}$
WDR5	7.43	6.68	12.7	8.60	13.8
D172A	9.51	11.7	17.5	14.7	14.4
P216L	10.9	10.5	14.3	9.73	12.9
Y260H	5.81	5.15	26.4	7.68	10.1
F133L	3.90	3.62	ND*	5.28	7.40
S175L	10.9	14.4	14.9	15.6	4.97
S218F	6.83	6.63	8.25	8.05	9.92

ND* stands for not determined.

<u>Supplementary Table S12.</u> Quantitative comparisons between affinity data resulting from BLI and FP measurements in terms of the ratio of the normalized $K_{D\text{-}BLI}$ to the normalized $K_{D\text{-}FP}$. A normalized K_D of the binding interactions of a SET1$_{Win}$ ligand with a WDR5 mutant is the K_D measured for this interaction pair divided by the K_D value measured with the same SET1$_{Win}$ ligand interacting with the native WDR5 protein.

Protein	MLL2$_{Win}$	MLL3$_{Win}$	MLL4$_{Win}$	SETd1A$_{Win}$	SETd1B$_{Win}$
D172A	1.28	1.76	1.37	1.71	1.04
P216L	1.47	1.57	1.13	1.13	0.94
Y260H	0.78	0.77	2.09	0.89	0.73
F133L	0.52	0.54	ND*	0.61	0.54
S175L	1.47	2.15	1.18	1.82	0.36
S218F	0.92	0.99	0.65	0.94	0.72

ND* stands for not determined.

17. Supporting references.

(1) Dharmarajan, V.; Lee, J. H.; Patel, A.; Skalnik, D. G.; Cosgrove, M. S. Structural basis for WDR5 interaction (Win) motif recognition in human SET1 family histone methyltransferases. *J. Biol. Chem.* **2012**, *287*, 27275-27289.

(2) Imran, A.; Moyer, B. S.; Canning, A. J.; Kalina, D.; Duncan, T. M.; Moody, K. J.; Wolfe, A. J.; Cosgrove, M. S.; Movileanu, L. Kinetics of the multitasking high-affinity Win binding site of WDR5 in restricted and unrestricted conditions. *Biochem. J.* **2021**, *478*, 2145-2161.

(3) Mayse, L. A.; Imran, A.; Larimi, M. G.; Cosgrove, M. S.; Wolfe, A. J.; Movileanu, L. Disentangling the recognition complexity of a protein hub using a nanopore. *Nature Commun.* **2022**, *13*, 978.

(4) Zhang, P.; Lee, H.; Brunzelle, J. S.; Couture, J. F. The plasticity of WDR5 peptide-binding cleft enables the binding of the SET1 family of histone methyltransferases. *Nucleic Acids Res.* **2012**, *40*, 4237-4246.

(5) Forbes, S. A.; Beare, D.; Bindal, N.; Bamford, S.; Ward, S.; Cole, C. G.; Jia, M.; Kok, C.; Boutselakis, H.; De, T.; Sondka, Z.; Ponting, L.; Stefancsik, R.; Harsha, B.; Tate, J.; Dawson, E.; Thompson, S.; Jubb, H.; Campbell, P. J. COSMIC: High-Resolution Cancer Genetics Using the Catalogue of Somatic Mutations in Cancer. *Curr. Protoc. Hum Genet.* **2016**, *91*, 10.11.11-10.11.37.

(6) Forbes, S. A.; Beare, D.; Boutselakis, H.; Bamford, S.; Bindal, N.; Tate, J.; Cole, C. G.; Ward, S.; Dawson, E.; Ponting, L.; Stefancsik, R.; Harsha, B.; Kok, C. Y.; Jia, M.; Jubb, H.; Sondka, Z.; Thompson, S.; De, T.; Campbell, P. J. COSMIC: somatic cancer genetics at high-resolution. *Nucleic Acids Res.* **2017**, *45*, D777-d783.

(7) Tate, J. G.; Bamford, S.; Jubb, H. C.; Sondka, Z.; Beare, D. M.; Bindal, N.; Boutselakis, H.; Cole, C. G.; Creatore, C.; Dawson, E.; Fish, P.; Harsha, B.; Hathaway, C.; Jupe, S. C.; Kok, C. Y.; Noble, K.; Ponting, L.; Ramshaw, C. C.; Rye, C. E.; Speedy, H. E.; Stefancsik, R.; Thompson, S. L.; Wang, S.; Ward, S.; Campbell, P. J.; Forbes, S. A. COSMIC: the Catalogue Of Somatic Mutations In Cancer. *Nucleic Acids Res.* **2019**, *47*, D941-d947.

(8) Ali, A.; Veeranki, S. N.; Tyagi, S. A SET-domain-independent role of WRAD complex in cell-cycle regulatory function of mixed lineage leukemia. *Nucleic Acids Res.* **2014**, *42*, 7611-7624.

(9) Patel, A.; Vought, V. E.; Dharmarajan, V.; Cosgrove, M. S. A conserved arginine-containing motif crucial for the assembly and enzymatic activity of the mixed lineage leukemia protein-1 core complex. *J. Biol. Chem.* **2008**, *283*, 32162-32175.

(10) Zhao, J.; Chen, W.; Pan, Y.; Zhang, Y.; Sun, H.; Wang, H.; Yang, F.; Liu, Y.; Shen, N.; Zhang, X.; Mo, X.; Zang, J. Structural insights into the recognition of histone H3Q5 serotonylation by WDR5. *Science advances* **2021**, *7*.

(11) Tina, K. G.; Bhadra, R.; Srinivasan, N. PIC: Protein Interactions Calculator. *Nucleic Acids Res.* **2007**, *35*, W473-476.
